# Systematic review with meta‐analysis: the accuracy of serological tests to support the diagnosis of coeliac disease

**DOI:** 10.1111/apt.16729

**Published:** 2022-01-18

**Authors:** Athena L. Sheppard, Martha M. C. Elwenspoek, Lauren J. Scott, Victoria Corfield, Hazel Everitt, Peter M. Gillett, Alastair D. Hay, Hayley E. Jones, Susan Mallett, Jessica Watson, Penny F. Whiting

**Affiliations:** ^1^ The National Institute for Health Research Applied Research Collaboration West (NIHR ARC West) at University Hospitals Bristol NHS Foundation Trust Bristol UK; ^2^ Population Health Sciences Bristol Medical School University of Bristol Bristol UK; ^3^ Primary Care Research Centre Faculty of Medicine University of Southampton Southampton UK; ^4^ Paediatric Gastroenterology Department Royal Hospital for Children and Young People Edinburgh UK; ^5^ Centre for Medical Imaging University College London London UK

**Keywords:** coeliac disease, diagnostic tests, meta‐analyses

## Abstract

**Background:**

There is growing support for a biopsy avoidant approach to diagnose coeliac disease in both children and adults, using a serological diagnosis instead.

**Aims:**

To assess the diagnostic accuracy of serological tests for coeliac disease in adults and children.

**Methods:**

Seven electronic databases were searched between January 1990 and August 2020. Eligible diagnostic studies evaluated the accuracy of serological tests for coeliac disease against duodenal biopsy. Risk of bias assessment was performed using QUADAS‐2. Bivariate random‐effects meta‐analyses were used to estimate serology sensitivity and specificity at the most commonly reported thresholds.

**Results:**

113 studies (n = 28,338) were included, all in secondary care populations. A subset of studies were included in meta‐analyses due to variations in diagnostic thresholds. Summary sensitivity and specificity of immunoglobulin A (IgA) anti‐tissue transglutaminase were 90.7% (95% confidence interval: 87.3%, 93.2%) and 87.4% (84.4%, 90.0%) in adults (5 studies) and 97.7% (91.0%, 99.4%) and 70.2% (39.3%, 89.6%) in children (6 studies); and of IgA endomysial antibodies were 88.0% (75.2%, 94.7%) and 99.6% (92.3%, 100%) in adults (5 studies) and 94.5% (88.9%, 97.3%) and 93.8% (85.2%, 97.5%) in children (5 studies).

**Conclusions:**

Anti‐tissue transglutaminase sensitivity appears to be sufficient to rule out coeliac disease in children. The high specificity of endomysial antibody in adults supports its use to rule in coeliac disease. This evidence underpins the current development of clinical guidelines for a serological diagnosis of coeliac disease. Studies in primary care are needed to evaluate serological testing strategies in this setting.

## Graphical abstract

Serological tests have good accuracy for diagnosing coeliac disease. Anti‐tissue transglutaminase sensitivity appears to be sufficient to rule out coeliac disease in children. The high specificity of endomysial antibody in adults supports its use to rule in coeliac disease.

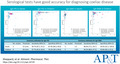

## 1 INTRODUCTION

Coeliac disease is a chronic small intestinal immune‐mediated enteropathy triggered by the ingestion of gluten, a protein found in wheat, rye and barley.^1^ Exposure to gluten results in intestinal damage of varying severity in patients affected by coeliac disease. Symptomatic coeliac disease is characterised by gastrointestinal symptoms, including diarrhoea, nausea, vomiting and abdominal pain, and extraintestinal symptoms such as fatigue and weight loss.

Coeliac disease is estimated to affect around 1% of people in the UK,^2^ however only 24% of those with coeliac disease are thought to be diagnosed.^3^ These large numbers of undiagnosed patients—known as the “coeliac iceberg”—are thought to be a consequence of the non‐specific nature of coeliac disease symptoms and variation in clinical presentation, from none (asymptomatic coeliac disease) to a broad spectrum of symptoms.^1^ People with certain health conditions, such as type I diabetes, autoimmune thyroid disease or Down syndrome, as well as first‐degree relatives of people with coeliac disease, are at higher risk of developing coeliac disease than the general population and are more likely to present without classical symptoms.^4^

Currently, the only treatment for coeliac disease is lifetime adherence to a gluten‐free diet, which is expensive and can be difficult to comply with. Left undiagnosed and untreated, coeliac disease often leaves patients with troublesome symptoms that significantly affect their quality of life and lead to a higher risk of complications such as osteoporosis, infertility and small bowel cancer.^5^ As such, a timely and accurate diagnosis of coeliac disease is important.

Coeliac disease is diagnosed using a combination of serological tests for coeliac‐specific antibodies and endoscopic intestinal biopsy. Current guidelines by the National Institute for Health and Care Excellence recommend both adults and children with suspected coeliac disease first undergo serological testing for total immunoglobulin A (IgA) and IgA anti‐tissue transglutaminase (tTG).^4^ In IgA deficient patients, immunoglobulin G (IgG) endomysial antibodies (EMA), IgG deamidated gliadin peptide (DGP) or IgG tTG can be used. In adults, weakly positive for IgA tTG, IgA EMA should be measured. Seropositive adults should be referred for intestinal biopsy, while seropositive children should be referred for further investigation, which may include intestinal biopsy, IgA EMA, human leukocyte antigen (HLA) genetic testing, or a combination of the above.^4^

Intestinal biopsy is invasive and can be burdensome for patients, particularly children, who require general anaesthesia to undergo the procedure. Patients must consume a gluten‐containing diet for at least six weeks prior to any serological test or biopsy, meaning those with coeliac disease may continue to experience painful and debilitating symptoms while they wait. Guidelines in the UK have begun to move towards biopsy‐avoidance strategies for coeliac disease in children and, more recently, in adults. In their 2013 guidelines, the British Society of Paediatric Gastroenterology, Hepatology and Nutrition advised that children with IgA tTG greater than or equal to 10x the upper limit of normal for the assay, positive for IgA EMA and HLA positive do not need to undergo biopsy to confirm their coeliac disease diagnosis.^6^ During the coronavirus pandemic, the British Society of Gastroenterology published interim guidance including a COVID‐19 specific non‐biopsy protocol for adults with suspected coeliac disease.^7^

Previous systematic reviews of the accuracy of serological testing for diagnosing coeliac disease suggest that the tests are highly sensitive and specific in both adults and children.^8^, ^9^, ^10^, ^11^, ^12^ These systematic reviews, however, are all out‐of‐date and most have methodological limitations. Limitations included: limited search^8^, ^9^, ^10^, ^11^; use of the Moses‐Littenberg model^13^ to pool estimates of sensitivity and specificity rather than the more robust bivariate or hierarchical summary receiver operating characteristic (HSROC) models^14^, ^15^ or no statistical synthesis of results^10^; and use of the original QUADAS‐tool^16^ to assess study quality (although at the time this was the most appropriate tool) the results of which were then not incorporated into the synthesis,^8^, ^9^, ^11^ or no quality assessment.^10^ The most recent, comprehensive review by Maglione et al, conducted for the AHRQ programme, was the only review to include more than 20 studies.^12^ However, this review also included existing systematic reviews and only generated overall summary estimates for studies published since existing reviews; it did not produce overall estimates of the accuracy of the included serological tests. This review was restricted to studies that either included at least 300 participants or were conducted in an “at risk” population—reasons for the sample size restriction were not justified. None of the reviews considered the study threshold when calculating pooled estimates of sensitivity and specificity.

The purpose of this systematic review is to provide a robust and up‐to‐date evaluation of the accuracy of serological tests for coeliac disease in adults and children.

## 2 MATERIALS AND METHODS

This review followed Cochrane recommended methods and guidance from the Centre for Reviews and Dissemination for systematic reviews of diagnostic test accuracy.^17^, ^18^ Our findings are reported in accordance with the PRISMA‐DTA guidelines.^19^ We developed and followed a standard protocol for all stages of the review, which was registered with PROSPERO (registration number: CRD42019115506).^20^ Any deviations from the protocol are indicated.

### 2.1 Literature search

MEDLINE, Embase, Cochrane Library, KSR Evidence and the Science databases on Web of Science were searched for relevant studies from January 1990 (when IgA EMA antibodies were introduced into practice) to August 2020, combining terms for “antibodies” and “coeliac disease” (see Appendix 1 for full strategies). Ongoing and completed studies were identified using the WHO International Clinical Trials Registry and the National Institutes of Health Clinical Trials database. Internet searches using keywords such as “celiac”/“coeliac” and “serological tests” were undertaken. The reference lists of relevant systematic reviews identified during the literature search were also used as a source of potentially relevant studies. No language restrictions were applied.

### 2.2 Inclusion criteria

Inclusion criteria were defined during protocol development and piloted on a subset of 500 articles at title and abstract screening to ensure functionality.

Studies using a diagnostic cohort design were included. Studies using a case‐control design were excluded as they have been shown to overestimate test accuracy and a substantial evidence base from cohort studies was anticipated.^21^

Studies in patients with classical symptoms of coeliac disease (eg, diarrhoea, abdominal pain, fatigue), as well as mixed symptomatic and risk group (eg, type I diabetic) populations, were included. After piloting our inclusion criteria, we chose to exclude studies in healthy individuals (ie, screening) or specific risk groups only to ensure the review was conducted in a clinically relevant population and that accuracy measures could be reasonably combined in a meta‐analysis.

Studies in which patients underwent at least one serological test for coeliac disease, including IgA tTG, IgG tTG, IgA EMA, IgG EMA, IgA DGP, IgG DGP and IgA anti‐actin antibodies (AAA), were included. Combined serological tests, such as IgA/IgG tTG (which detect the presence of IgA tTG or IgG tTG in a serum sample) were also included.

Studies were included if the diagnosis was confirmed by duodenal biopsy and if at least some seronegative patients also underwent a biopsy. Studies in which serology formed part of the reference standard, which could lead to overestimation of accuracy, were excluded.

### 2.3 Methods of study selection, data extraction and quality assessment

#### 2.3.1 Study selection

Titles and abstracts identified through electronic database and web searching were uploaded to Rayyan and independently screened by two reviewers (ALS, MMCE or VC).^22^ Articles considered potentially relevant were obtained and assessed by one reviewer (ALS) and checked by a second reviewer (MMCE, LJS or VC) for inclusion in the review. Any discrepancies between reviewers were resolved through discussion or referral to a third reviewer.

#### 2.3.2 Data extraction

Data from each study were extracted by one reviewer (ALS) and all were checked by a second (MMCE, LJS or VC) using data extraction forms developed in Microsoft Access 2016. Disagreements were resolved through discussion or referral to a third reviewer. Data on study and patient characteristics, serological tests, and biopsy procedures were extracted.

Two‐by‐two data comparing serological test results with reference standard (biopsy) results (number of true positives, false negatives, false positives and true negatives) were extracted. Data relating to patients that did not undergo biopsy were excluded from the 2 × 2 tables where possible. Where 2 × 2 data were reported at multiple thresholds within a study, data relating to the manufacturer or study authors’ pre‐specified cut‐off were extracted. Where a threshold of primary importance was not pre‐specified, data relating to the lowest reported threshold were extracted. Two‐by‐two data were extracted at biopsy cut‐off Marsh Grade 3a if available, or at any reported biopsy cut‐off otherwise.

#### 2.3.3 Study quality

Included studies were assessed for methodological quality using the QUADAS‐2 tool,^23^ tailored to our review (Appendix 2), which evaluates the risk of bias and applicability in primary diagnostic accuracy studies. The tool consists of four domains: patient selection, index test, reference standard, and flow and timing, each rated as high, low or unclear risk of bias. If at least one of the domains was rated as “high,” the study was considered at high risk of bias; if all domains were judged as “low” the study was considered at low risk of bias; otherwise, the study was considered as “unclear” risk of bias.

When a study reported accuracy data for two or more tests, the “index test” and “flow and timing” domains were applied separately to each test. When a study reported accuracy data for adults and children separately, all domains were applied separately to each patient group.

### 2.4 Quantitative analysis and meta‐analysis methods

Analyses were stratified by age group (adults >16 years; children ≤16 years; mixed [adults and children] and age unspecified) and test. All analyses were performed in Stata version 16.0 using the metandi command.^24^

#### 2.4.1 Primary analyses

For data sets including four or more studies, a bivariate random‐effects meta‐analysis of sensitivity and specificity was performed,^14^ assuming binomial likelihoods for the number of true positive and true negative test results.^25^ When there were few (2‐3) studies in a data set, univariate fixed‐effect meta‐analyses of sensitivity and specificity were performed. Where only a single study was available, the sensitivity and specificity reported in that study are presented.

Where the extracted data on a test related to a range of thresholds, we report results from two separate meta‐analyses. First, we fitted the bivariate model^14^ to studies reporting at the most commonly reported threshold only. From these models, we report summary sensitivity and specificity at that threshold. We used summary estimates of sensitivity and specificity to calculate summary positive and (inverse) negative likelihood ratios and associated confidence intervals. Second, we fitted the HSROC model^15^ to the full data set, which consisted of one estimate per study to avoid double counting. From these models, we present the summary receiver operating characteristic (ROC) curve, which represents the trade‐off between sensitivity and specificity across thresholds.

The sensitivity and specificity reported in each study were plotted in ROC space, with colour coding allowing for comparisons between different thresholds to be made. Summary estimates of sensitivity and specificity with 95% confidence intervals at the most commonly reported threshold and summary ROC curves across all reported thresholds are presented.

Summary positive and negative predictive values and natural frequencies were estimated for a hypothetical population of 10,000 people tested for coeliac disease, for a pre‐test probability of 2% (the estimated pre‐test probability of coeliac disease in a primary care population presenting with symptoms suggestive of coeliac disease^26^). Values were estimated based on summary sensitivity and specificity, restricted to the most commonly reported threshold.

#### 2.4.2 Direct comparisons

For the two most commonly assessed tests, IgA tTG and IgA EMA, we also estimated the relative sensitivity and specificity within each study to summarise their comparative accuracy. Relative sensitivity is a ratio of two sensitivities, for example if relative sensitivity is 1 then the sensitivity of the two tests is the same (similarly for specificity). We had intended to pool estimates of relative sensitivity and specificity. However, none of the studies that evaluated comparative accuracy reported estimates of sensitivity and specificity for the same thresholds. We therefore report the observed range of these measures across comparative studies (which evaluated both tests in the same group of patients). The relative accuracy of tests with a high estimated sensitivity and/or specificity (>90% across all studies), that were compared to IgA tTG or IgA EMA, are also reported.

#### 2.4.3 Sensitivity analyses

Sensitivity analyses were performed restricting inclusion to: (1) studies rated at low risk of bias using the QUADAS‐2 tool, (2) studies carried out in symptomatic patients only and (3) studies in which all patients received a biopsy.

### 2.5 Patient and public involvement

Patients and the public were not involved in the choice of research question, the design of the study, the conduct of the study, the interpretation of the results, or our dissemination plans.

### 2.6 Deviations from the protocol

In the protocol for this review,^20^ we described our target population as “adults or children at risk of coeliac disease.” After piloting out inclusion criteria at title and abstract screening, we chose to exclude studies in healthy populations (ie, screening) or single risk groups only, as described in the Inclusion criteria.

We described the intervention as “any serological test for coeliac disease,” including HLA‐DQ typing. We decided not to include anti‐gliadin antibodies as they are not recommended for use in the diagnosis of coeliac disease by the National Institute for Health and Care Excellence.^4^ We decided to focus this review on serological tests; we have evaluated the accuracy of HLA testing in a separate review.^26^ We did not include point‐of‐care or rapid serological tests as a systematic review of their accuracy has recently been published.^27^

We described our comparator as “any reported reference standard.” After piloting our exclusion criteria at title and abstract screening, we decided to exclude studies where serology formed part or all of the reference standard as this would lead to over‐inflation of test accuracy estimates.

In the strategy for data synthesis, we said “If a test is reported at a single threshold for test positivity across studies, summary operating points will be used to measure the test's accuracy. If a test is reported at differing thresholds across studies, summary ROC curves showing the trade‐off between sensitivity and specificity at the various thresholds will be produced.” In the review, we produced both summaries of the evidence for completeness: a summary ROC curve across all reported thresholds and summary sensitivity/specificity at the most commonly reported threshold.

## 3 RESULTS

### 3.1 Study characteristics

**FIGURE 1:**
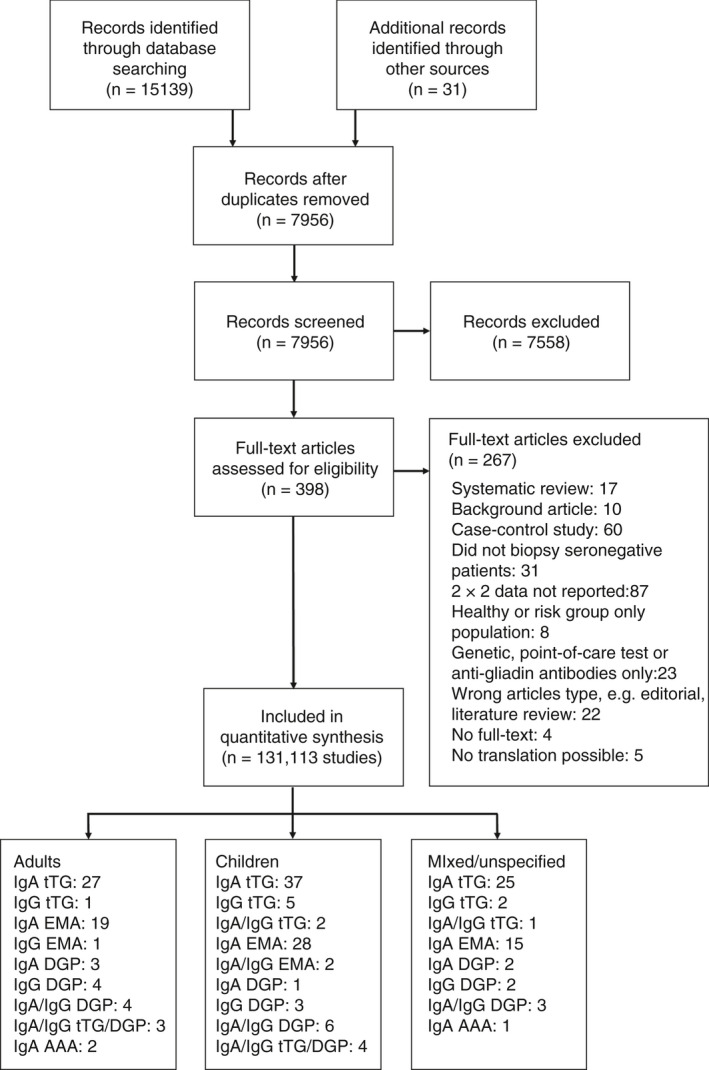
PRISMA diagram showing the flow of studies through the review. Abbreviations: IgA, immunoglobulin A; IgG, immunoglobulin G; tTG, anti‐tissue transglutaminase; EMA, endomysial antibodies; DGP, deamidated gliadin peptide; AAA, anti‐actin antibodies

A total of 15 170 articles were identified through electronic searches (see PRISMA study flow diagram in Figure 1). After removing duplicates, the titles and abstracts of 7956 articles were independently screened by two reviewers, of which 398 were considered potentially relevant and full‐texts were obtained. We were unable to obtain full‐texts for four studies and translation was not possible for a further five studies. After further assessment for eligibility, 113 studies in 131 publications fulfilled our inclusion criteria. Two hundred and three sets of 2 × 2 data on a total of 28 338 patients were extracted across the studies.

Twenty‐nine studies were conducted in adults, 48 in children and 33 in a mixed (adults and children) population or an age unspecified population.^1^ A further three studies reported separate accuracy data for adults and children, which were extracted as two distinct sets of data. Where reported, the mean age of adults was 43.6 years (standard deviation: 13.9 years, range: 13‐94 years) and of children was 6.3 years (standard deviation: 4.4 years, range: 2 months‐19 years). On average 66% of adults and 52% of children were female. Fifty‐six studies were prospective and 57 were retrospective in design. See Appendix 3 for full characteristics of included studies.

### 3.2 Quality of evidence

One hundred and thirty‐seven sets of 2 × 2 data were judged to be at high risk of bias, 22 were low risk of bias and 44 were deemed unclear (Appendix 4).

Most (118) sets of 2 × 2 data were at high risk of bias because biopsy results were interpreted with knowledge of (or not explicitly blinded to) serology results. In 28 sets of 2 × 2 data, there was potential for partial verification bias due to some patients not undergoing biopsy to verify their true disease status. A further 23 sets of 2 × 2 data were rated high risk of bias due to concerns about patient selection (eg, inappropriate study exclusions, patients not adhering to a gluten‐free diet prior to testing) and 12 due to concerns about the index test (eg, threshold not pre‐specified).

Twenty‐four sets of 2 × 2 data were judged to be at unclear risk of bias due to missing information on patient selection (eg, study exclusion criteria), 23 where details of serological testing (eg, threshold for test positivity) were not reported and 40 where information on flow and timing (eg, interval between serology and biopsy, or whether patients maintained a gluten‐free diet between tests) was not reported.

### 3.3 Primary analyses

#### 3.3.1 All thresholds

All accuracy data extracted from the included studies are summarised in Table 1. IgA tTG and IgA EMA were the most commonly studied tests across all age groups, with 27 and 37 studies of IgA tTG and 19 and 28 studies of IgA EMA performed in adults and children, respectively. Coeliac disease prevalence varied greatly between studies (range: 1.8%‐92.6%).

**TABLE 1:** Study estimates of test accuracy, stratified by age group and test

Serological test	Studies, n	Participants (coeliac disease), n	Threshold	Sensitivity (range), %	Specificity (range), %
All data					
Adults					
IgA tTG	27	11 355 (2566)	5‐25 U/mL	35.2‐100.0	0.0‐100.0
IgG tTG	1	65 (14)	10 U/mL	71.4	96.1
IgA EMA	19	7122 (1028)	1:5‐1:20	61.3‐100.0	87.5‐100.0
IgG EMA	1	96 (28)	1:20	39.3	98.5
IgA DGP	3	885 (154)	10‐20 U/mL	85.7‐98.3	92.2‐95.7
IgG DGP	4	1046 (217)	10‐20 U/mL	90.0‐96.7	99.2‐100.0
IgA/IgG DGP	4	1161 (280)	20 U/mL	86.2‐98.3	95.9‐98.8
IgA/IgG tTG/DGP	3	1849 (173)	20 U/mL	72.2‐96.3	80.4‐97.4
IgA AAA	2	820 (140)	25 U/mL	80.0, 86.7	92.2, 95.1
Children					
IgA tTG	37	7944 (4164)	3‐100 U/mL	28.6‐100.0	7.9‐100.0
IgG tTG	5	599 (278)	3, 7 U/mL	31.0‐97.4	70.8‐100.0
IgA/IgG tTG	2	742 (282)	6, 45.1 U/mL	94.4, 96.0	85.8, 99.5
IgA EMA	28	4974 (2472)	1:5‐1:40	40.0‐100.0	29.4‐100.0
IgA/IgG EMA	2	173 (131)	1:2.5, 1:5	95.3, 95.7	74.2, 90.9
IgA DGP	1	212 (109)	20 U/mL	85.3	88.3
IgG DGP	3	1135 (669)	10‐25 U/mL	77.2‐92.0	83.5‐94.1
IgA/IgG DGP	6	941 (464)	16‐20 U/mL	87.5‐100.0	22.2‐96.4
IgA/IgG tTG/DGP	4	986 (415)	3‐32.7 U/mL	87.5‐98.2	61.2‐99.5
Mixed or unspecified					
IgA tTG	25	4564 (1414)	2‐89.5 U/mL	38.1‐100.0	9.5‐100.0
IgG tTG	2	432 (122)	10, 18.9 U/mL	40.6, 84.6	78.0, 89.0
IgA/IgG tTG	1	254 (26)	7.8 U/mL	92.3	82.9
IgA EMA	15	2884 (843)	1:2.5‐1:10	68.0‐100.0	38.9‐100.0
IgA DGP	2	561 (58)	19.9 U/mL	77.1, 90.0	93.4, 96.6
IgG DGP	2	562 (56)	19.9 U/mL	76.1, 80.0	92.0, 99.2
IgA/IgG DGP	3	480 (48)	NR	70.6‐85.7	92.9‐98.7
IgA AAA	1	391 (10)	NR	50	91.3

Abbreviations: AAA, anti‐actin antibodies; DGP, deamidated gliadin peptide; EMA, endomysial antibodies; IgA, immunoglobulin A; IgG, immunoglobulin G; NR, not reported; tTG, anti‐tissue transglutaminase.

**FIGURE 2:**
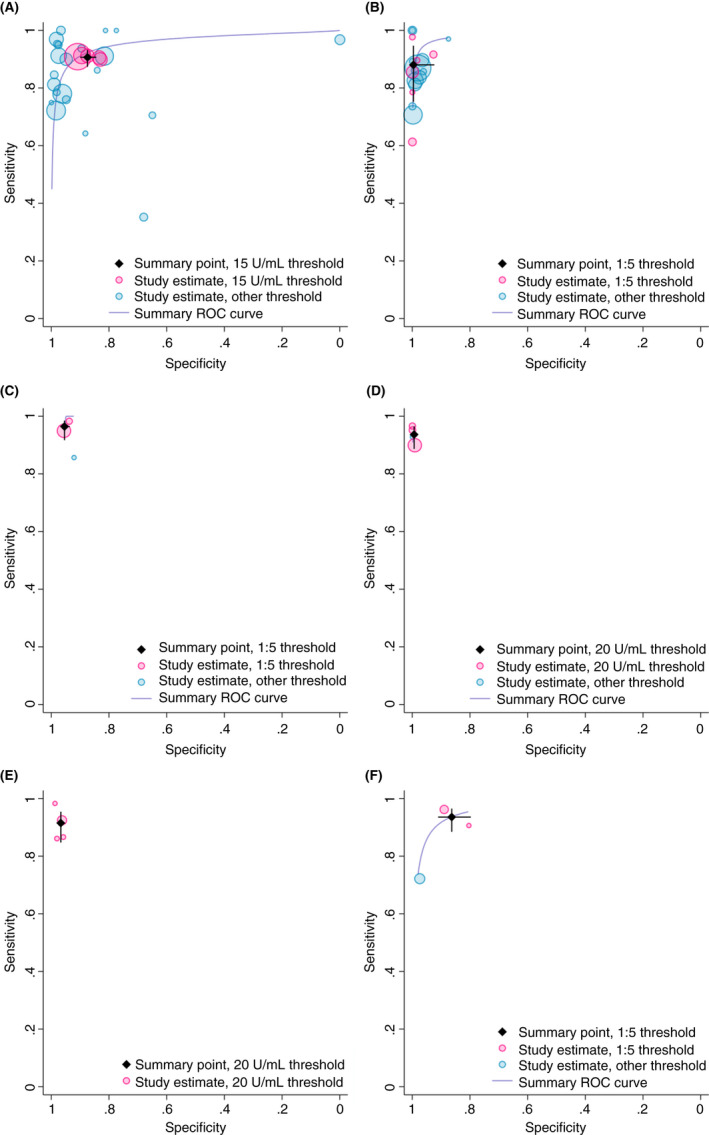
Study estimates of test sensitivity and specificity in adults plotted in receiver operating characteristic (ROC) space. Summary ROC curves are estimated from a meta‐analysis of all data, across thresholds. Summary point estimates are estimated from a meta‐analysis of data reporting at the most commonly reported threshold only. [A] Immunoglobulin A (IgA) anti‐tissue transglutaminase (tTG); [B] IgA endomysial antibodies; [C] IgA deamidated gliadin peptide (DGP); [D] Immunoglobulin G (IgG) DGP; [E] IgA/IgG DGP; [F] IgA/IgG tTG/DGP

**FIGURE 3:**
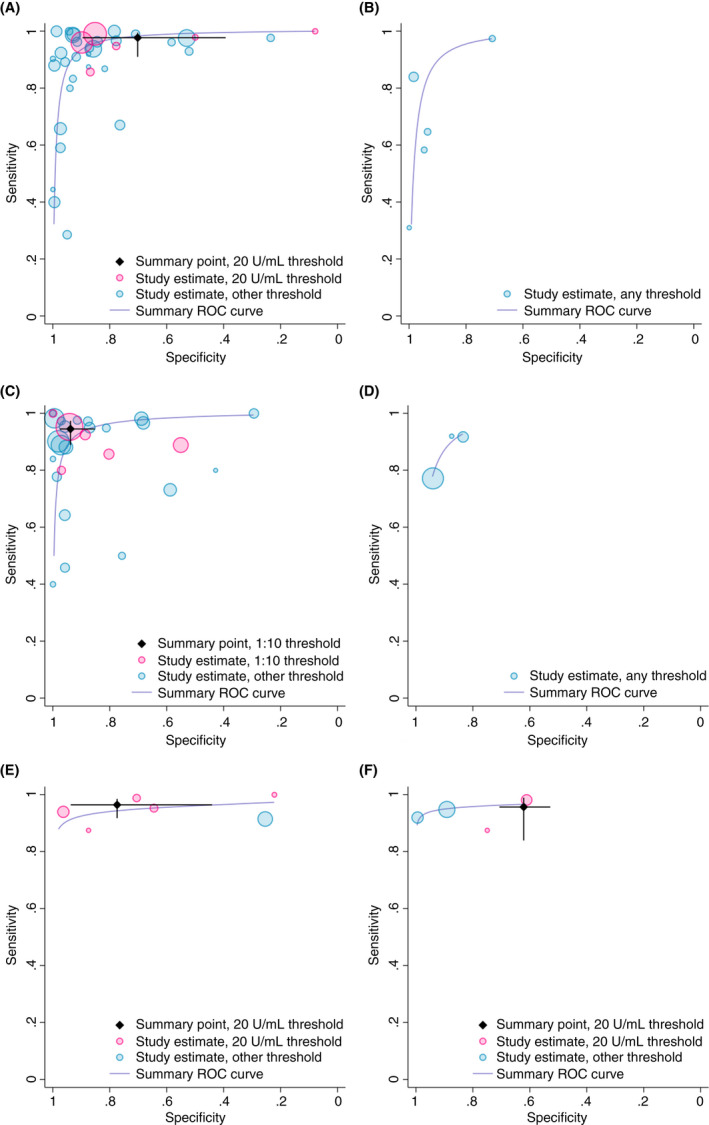
Study estimates of test sensitivity and specificity in children plotted in receiver operating characteristic (ROC) space. Summary ROC curves are estimated from a meta‐analysis of all data, across thresholds. Summary point estimates are estimated from a meta‐analysis of data reporting at the most commonly reported threshold only. [A] Immunoglobulin A (IgA) anti‐tissue transglutaminase (tTG); [B] Immunoglobulin G (IgG) tTG; [C] IgA endomysial antibodies; [D] IgG deamidated gliadin peptide (DGP); [E] IgA/IgG DGP; [F] IgA/IgG tTG/DGP

Study estimates of sensitivity and specificity are shown in ROC space in Figures 2 and 3, with summary ROC curves estimated across the full range of thresholds and summary estimates restricted to the most common threshold. There was considerable heterogeneity in sensitivity and specificity between studies.

#### 3.3.2 Most common threshold

At a threshold of 15 U/mL (5 studies), IgA tTG was moderately sensitive (90.7%, 95% confidence interval: 87.3, 93.2) and specific (87.4%, 84.4, 90.0) for coeliac disease in adults (Table 2). IgA EMA was highly specific (99.6%, 92.3, 100.0) in adults at a threshold of 1:5 (5 studies), but less sensitive (88.0%, 75.2, 94.7). IgA tTG was highly sensitive (97.7%, 91.0, 99.4) in children at a threshold of 20 U/mL (6 studies) but less specific (70.2%, 39.3, 89.6), while IgA EMA was both highly sensitive (94.5%, 88.9, 97.3) and specific (93.8%, 85.2, 97.5) at a threshold of 1:10 (5 studies). Using IgA EMA, adults and children with coeliac disease were 341x and 11x more likely to test positive for coeliac disease than patients without the condition, respectively (Table 2). Children without coeliac disease were 30x more likely to test negative on IgA tTG and 15x more likely to test negative on IgA EMA than patients with coeliac disease.

**TABLE 2:** Summary estimates of sensitivity, specificity and likelihood ratios, restricted to the most commonly reported threshold only

Serological test	Studies, n	Participants (coeliac disease), n	Threshold	Sensitivity (95% CI), %	Specificity (95% CI), %	Positive likelihood ratio (95% CI)	Inverse negative likelihood ratio (95% CI)
Most common threshold							
Adults							
IgA tTG	5	4310 (454)	15 U/mL	90.7 (87.3, 93.2)	87.4 (84.4, 90.0)	7.2 (5.8, 9.1)	9.4 (6.8, 12.8)
IgA EMA	5	927 (446)	1:05	88.0 (75.2, 94.7)	99.6 (92.3, 100.0)	340.7 (12.2, 9539.8)	7.6 (3.9, 14.7)
IgA DGP^†^	2	820 (140)	20 U/mL	96.4 (91.7, 98.5)	95.4 (93.6, 96.8)	21.2 (15.0, 29.9)	26.7 (11.3, 63.2)
IgG DGP^†^	3	981 (203)	20 U/mL	93.6 (88.6, 96.5)	99.4 (98.5, 99.7)	145.7 (60.8, 349.4)	15.6 (8.6, 28.3)
IgA/IgG DGP	4	1161 (280)	20 U/mL	91.5 (84.7, 95.4)	96.7 (95.3, 97.7)	27.7 (19.0, 40.5)	11.4 (6.2, 20.9)
IgA/IgG tTG/DGP^†^	2	851 (155)	20 U/mL	93.5 (88.4, 96.5)	86.3 (79.7, 91.0)	6.8 (4.5, 10.3)	13.4 (7.3, 24.5)
IgA AAA^†^	2	820 (140)	25 U/mL	82.9 (75.7, 88.2)	92.5 (90.3, 94.3)	11.0 (8.4, 14.5)	5.4 (3.7, 7.8)
Children							
IgA tTG	6	2232 (1051)	20 U/mL	97.7 (91.0, 99.4)	70.2 (39.3, 89.6)	3.3 (1.3, 8.0)	30.4 (8.5, 108.7)
IgA EMA	5	1257 (685)	1:10	94.5 (88.9, 97.3)	93.8 (85.2, 97.5)	11.2 (3.3, 37.4)	14.8 (7.7, 28.2)
IgA/IgG DGP	5	533 (276)	20 U/mL	96.4 (91.7, 98.5)	77.4 (44.0, 93.7)	4.3 (1.4, 13.1)	21.5 (10.7, 43.4)
IgA/IgG tTG/DGP^†^	2	244 (133)	20 U/mL	95.6 (83.9, 98.9)	62.2 (52.8, 70.7)	2.5 (2.0, 3.2)	14.3 (3.6, 57.0)

Abbreviations: AAA, anti‐actin antibodies; CI, confidence interval; DGP, deamidated gliadin peptide; EMA, endomysial antibodies; IgA, immunoglobulin A; IgG, immunoglobulin G; tTG, anti‐tissue transglutaminase.

^†^ Univariate fixed‐effect meta‐analysis.

**FIGURE 4:**
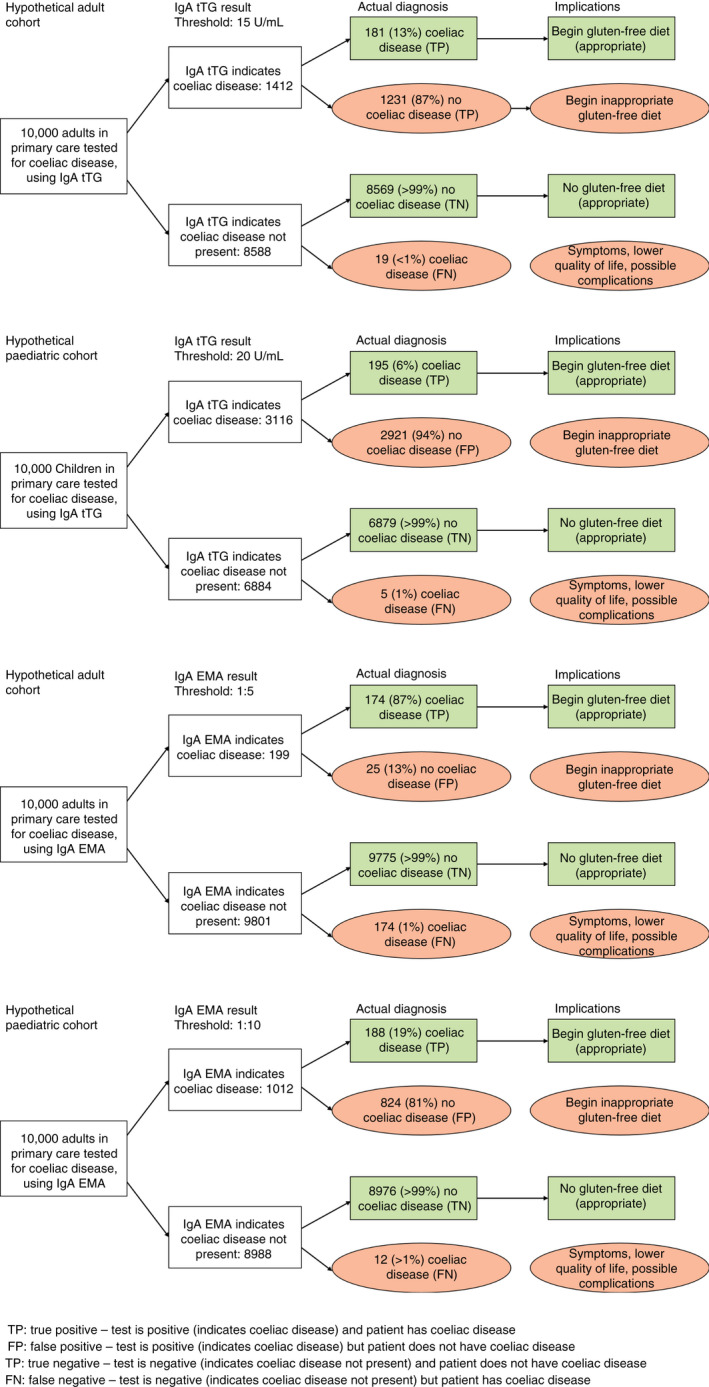
Diagram showing results that would be obtained if a hypothetical cohort of 10,000 adults and children were tested for coeliac disease using immunoglobulin A (IgA) anti‐tissue transglutaminase (tTG) or IgA endomysial antibodies (EMA), assuming a prevalence of 2%

Figure 4 shows the results that would be obtained if a hypothetical cohort of patients who present to primary care with symptoms suggestive of coeliac disease (pre‐test probability of 2%^26^) were tested for coeliac disease. A high number of false‐positive results are likely to be observed if IgA tTG or IgA EMA were used in isolation at the most commonly reported thresholds (Figure 4).

### 3.4 Sensitivity analyses

There was little evidence that estimates of sensitivity and specificity varied according to study quality, whether all patients presented with symptoms or whether all patients within a study underwent biopsy (Table 3 and Appendix 5). However, formal comparison was not possible as too few studies within each subgroup reported accuracy estimates at consistent thresholds.

**TABLE 3:** Study estimates of sensitivity and specificity limited to specific subgroups, stratified by age group and test

Serological test	Studies, n	Participants (coeliac disease), n	Threshold	Sensitivity (range), %	Specificity (range), %
Symptomatic patients only					
Adults					
IgA tTG	7	4244 (325)	5‐20 U/mL	64.3‐100.0	88.2‐98.1
IgA EMA	8	3786 (327)	1:5‐1:20	61.3‐100.0	98.1‐100.0
Children					
IgA tTG	8	1126 (615)	4‐20 U/mL	40.0‐100.0	7.9‐100.0
IgA EMA	8	1327 (753)	1:5‐1:20	40.0‐97.6	42.9‐100.0
All patients underwent biopsy					
Adults					
IgA tTG	26	11 183 (2444)	5‐25 U/mL	64.3‐100.0	0.0‐100.0
IgA EMA	18	7010 (1021)	1:5‐1:20	61.3‐100.0	87.5‐100.0
Children					
IgA tTG	31	5824 (3330)	3‐100 U/mL	28.6‐100.0	23.5‐100.0
IgA EMA	22	3685 (2015)	1:5‐1:40	45.8‐100.0	29.4‐100.0
Low risk of bias					
Adults					
IgA tTG	5	1577 (426)	5‐20 U/mL	76.0‐100.0	87.5‐98.3
IgA EMA	2	268 (103)	1:5, 1:20	61.3, 89.3	100.0, 100.0
Children					
IgA tTG	4	1319 (823)	20‐21 U/mL	94.8‐100.0	23.5‐98.7
IgA EMA	1	873 (528)	1:10	95.3	94.2

Abbreviations: EMA, endomysial antibodies; IgA, immunoglobulin A; tTG, anti‐tissue transglutaminase.

### 3.5 Direct comparisons

Comparative accuracy studies provided little evidence of differences in accuracy between tests (Table 4). Fourteen studies in adults and 16 studies in children provided direct comparison of IgA tTG and IgA EMA. There was a suggestion that IgA EMA was more specific than IgA tTG in adults with similar estimates of sensitivity; estimates in children were similar for both tests. However, studies reported results at different thresholds therefore formal statistical comparison was not appropriate.

**TABLE 4:** Study estimates of sensitivity and specificity, restricted to comparative studies only

Serological test	Studies, n	Participants (coeliac disease), n	Threshold	Sensitivity (range), %	Relative sensitivity (range), %	Specificity (range), %	Relative specificity (range), %
Comparative data							
Adults							
IgA tTG vs IgA EMA	14	6575 (881)					
IgA tTG			5‐25 U/mL	64.3‐100.0		81.3‐99.1	
IgA EMA			1:05	61.3‐100.0	0.81‐1.22	87.5‐100.0	1.00‐1.17
IgA tTG vs IgA DGP	3	885 (154)					
IgA tTG			10‐20 U/mL	64.3‐95.0		88.2‐97.5	
IgA DGP			10‐20 U/mL	85.7‐98.3	1.04‐1.33	92.2‐95.7	0.96‐1.04
IgA tTG vs IgG DGP	4	1046 (217)					
IgA tTG			10‐20 U/mL	64.3‐95.2		88.2‐98.0	
IgG DGP			10‐20 U/mL	90.0‐96.7	0.99‐1.44	99.2‐100.0	1.02‐1.13
IgA tTG vs IgA/IgG DGP	4	1161 (280)					
IgA tTG			5‐20 U/mL	76.0‐95.0		94.8‐99.0	
IgA/IgG DGP			20 U/mL	86.2‐98.3	1.01‐1.14	95.9‐98.8	0.99‐1.01
Children							
IgA tTG vs IgA EMA	16	3021 (1746)					
IgA tTG			5.5‐21 U/mL	28.6‐99.0		23.5‐100.0	
IgA EMA			1:5‐1:10	50.0‐100.0	0.91‐2.25	29.4‐100.0	0.76‐1.25

Abbreviations: DGP, deamidated gliadin peptide; EMA, endomysial antibodies; IgA, immunoglobulin A; IgG, immunoglobulin G; tTG, anti‐tissue transglutaminase.

Other test pairs were only compared directly in three or four studies. IgG DGP and IgA/IgG DGP appeared slightly more sensitive and specific than IgA tTG, however, this difference was much smaller than indirect comparisons suggested (Table 2). This suggests that studies providing a direct comparison between DGP and other serological tests may be subject to bias, resulting in overestimated accuracy for all tests evaluated in these studies.

## 4 DISCUSSION

### 4.1 Statement of principal findings

The accuracy of serological tests for detecting coeliac disease was high. IgA tTG was found to have a sensitivity of 90.7% (95% confidence interval: 87.3, 93.2, threshold: 15 U/mL) in adults and 97.7% (91.0, 99.4, threshold: 20 U/mL) in children, based on five and six studies, respectively. Specificity was slightly lower at 87.4% (84.4, 90.0) in adults and 70.2% (39.3, 89.6) in children. This suggests that IgA tTG is better at ruling out a diagnosis of coeliac disease than at ruling in coeliac disease.

The sensitivity of IgA EMA was slightly lower at 88.0% (75.2, 94.7, threshold: 1:5) in adults and 94.5% (88.9, 97.3, threshold: 1:10) in children based on five studies each. Specificity was higher at 99.6% (92.3, 100.0) in adults and 93.8% (85.2, 97.5) in children. This suggests that EMA may be useful in ruling in coeliac disease, possibly as a secondary test following an initial positive IgA tTG test. However, there were insufficient data to formally evaluate its use as an add‐on test.

### 4.2 Strengths and limitations

There are several key strengths to this review, which avoids the methodological limitations highlighted in previous reviews. Limiting inclusion to diagnostic cohort studies helps to ensure the quality of the supporting evidence and avoids overestimation of test accuracy due to potential bias introduced by case‐control designs. However, this also means that fewer studies were available to contribute to summary estimates, potentially resulting in reduced precision of these estimates. Potentially relevant studies were identified through an extensive literature search and screening was carried out independently by two reviewers at each stage. We identified 113 studies that fulfilled our review inclusion criteria, considerably more than were included in previous reviews; the largest number of studies included in any of the previous reviews identified was 31 studies in addition to 11 reviews that included smaller numbers of studies.^12^ Data extraction was also performed by one reviewer and checked by a second to ensure accuracy and completeness. We conducted a detailed risk of bias assessment using an appropriate and validated tool.^23^ Syntheses of studies were carried out in line with Cochrane recommended methods and sensitivity analyses were performed to explore heterogeneity.^17^, ^18^

A large amount of heterogeneity was present across included studies. A wide variety of thresholds for test positivity were reported across studies, with some not reporting the threshold at all. There is a lack of clarity on how thresholds relate to one another across laboratories and manufacturers. Where threshold units differed between assays we assumed they represented the same arbitrary units and were comparable, however as they do not measure absolute amounts of antibodies there may be a slight variation between different commercial assays. We would have liked to investigate differences between commercial kits and whether the accuracy of tests has changed over time as new testing methods have evolved. However, the different thresholds at which results were reported and wide variety of commercial kits employed mean that there were insufficient data to allow us to stratify our analysis in this way.

There was substantial variation in coeliac disease prevalence between studies, likely due to differences in patient characteristics such as clinical presentation and reason for biopsy. Some studies excluded patients with IgA deficiencies while others did not, which may have affected the accuracy estimates for tests that detected the presence of IgA in serum samples. Sources of heterogeneity were explored through sensitivity analyses, but summary results were relatively robust to a number of exclusions.

All studies were carried out in a secondary care population by nature of the reference standard (duodenal biopsy), which limits the generalisability of the review. Caution is needed if trying to extrapolate these results to testing outside of hospital clinics in community populations where prevalence is lower and thus the probability that a positive result is false increases. There was some variability in biopsy procedure across studies; where biopsies were collected in an optimal manner this may have impacted study specificity.

Despite limiting our review to cohort studies, most sets of 2 × 2 data (67%) included were judged to be at high risk of bias, mainly due to a lack of blinding to serology when interpreting biopsy results. Partial verification bias may be present where not all patients in a study underwent biopsy, whether due to study design, clinical practice or deviation from study protocol. A recent systematic review suggests that failure to account for partial verification bias may result in overestimated sensitivity of IgA tTG.^28^ However, our sensitivity analysis of sets of 2 × 2 data at low risk of bias found little difference in test accuracy estimates compared to the evidence base as a whole, however, there was only a small pool of data on which to base this comparison and formal statistical comparison was not possible.

### 4.3 Comparison to existing literature

Existing evidence on the accuracy of serology for diagnosing coeliac disease is mixed. In a systematic review of serological test accuracy, Rostom et al stratified their analyses by age, test and substrate.^8^ Summary sensitivity and specificity of IgA tTG (human recombinant) and IgA EMA were >90% across all age groups. Giersiepen et al conducted a systematic review of antibody test accuracy in children.^9^ Meta‐analyses were not performed due to between‐study heterogeneity; sensitivity and specificity of IgA tTG ranged from 13% to 100% and 78% to 100%, and of IgA EMA from 83% to 100% and 95% to 100%. Schyum and Rumessen carried out a systematic review of serological test accuracy in adults.^10^ Study data was not meta‐analysed, but median sensitivity and specificity of IgA tTG were estimated as 93% and 95%, and of IgA EMA as 84% and 100%. van der Windt et al estimated serological test accuracy in adults presenting with abdominal symptoms in primary care.^11^ Summary sensitivity and specificity were 89% and 98% for IgA tTG and 90% and 99% for IgA EMA. Sensitivity and specificity estimates of IgA tTG and IgA EMA in this review were slightly lower than in previous reviews. The inclusion of case‐control studies may have inflated previous accuracy estimates.

### 4.4 Implications for clinical practice and future research

Serological tests are useful as a first step towards diagnosis in patients with suspected coeliac disease. The British Society of Paediatric Gastroenterology, Hepatology and Nutrition guidelines have already incorporated the safe and secure serological diagnosis of coeliac disease, allowing children meeting certain criteria—including IgA tTG ≥10× the upper limit of normal across a number of different assays—to be diagnosed without biopsy. These guidelines have since been validated in large prospective studies.^29^, ^30^ IgA tTG accuracy should always be internally validated against biopsy results within a practice, due to variation between assays and laboratory procedures.

There is increasing evidence of the high predictive value of IgA tTG ≥10× the upper limit of normal in an adult population,^31^ with interim guidance including a non‐biopsy protocol for adults with suspected coeliac disease published in light of the coronavirus pandemic.^7^ We found IgA EMA to be highly specific in adults, lending support to its utility as a secondary test to reduce the likelihood of a false positive tTG result. This may help to pave the way for serological diagnosis of coeliac disease in an adult population in the future, a topic that is of great interest to the gastroenterology community. Although, EMA is not available in all labs because it depends much more on observer interpretation than other tests such as tTG.

The interpretation of serological test results remains an important area of research, and further work is needed to confirm the thresholds above and below which we can confidently rule in or rule out coeliac disease. The practice of dichotomising continuous test results may be an oversimplification of a complex disease with a wide range of clinical presentations. Further research is also needed to estimate the accuracy of serological tests used in sequence or combination and to model the clinical and cost effectiveness of tests and testing strategies. Identification of highly accurate serological testing strategies may allow for progressively more biopsy‐avoidant pathways in the future. To assess the accuracy of a serological diagnosis in adults, an analysis of IgA tTG at the full range of thresholds should be undertaken to establish a cut‐off for which the positive predictive value is close to 100%. There are further tests in development for coeliac disease that will require evaluation, with research focussed on rapid point‐of‐care tests and genetic tests such as the HLA‐DQ‐gluten tetramer test.^32^

There is a need for research on serological test accuracy in primary care settings where serological tests are used in practice. With the growing movement towards biopsy‐avoidant pathways, the diagnosis and management of coeliac disease is likely to increasingly take place in primary rather than secondary care. It is therefore key that serological testing strategies are evaluated in primary care populations.

## AUTHORSHIP

*Guarantor of the article*: Athena L Sheppard.

*Author contributions:* Conceptualisation: ALS, MMCE, LJS, VC, HE, PMG, HEJ, JW, PFW. Data curation: ALS, MMCE, LJS, VC. Formal analysis: ALS, MMCE, LJS, HE, PMG, ADH, HEJ, SM, JW, PFW. Funding acquisition: PFW. Investigation: ALS, MMCE, LJS, HE, PMG, ADH, HEJ, SM, JW, PFW. Methodology: ALS, MMCE, LJS, HE, PMG, ADH, HEJ, SM, JW, PFW. Project administration: ALS. Resources: ALS. Software: ALS. Supervision: PFW. Validation: ALS, MMCE, LJS, HE, PMG, ADH, HEJ, SM, JW, PFW. Visualisation: ALS, MMCE, LJS, HE, PMG, ADH, HEJ, SM, JW, PFW. Writing—original draft: ALS. Writing—review and editing: ALS, MMCE, LJS, VC, HE, PMG, ADH, HEJ, SM, JW, PFW. All authors approved the final version of the manuscript.

*Declaration of funding interests:* This project was funded by the National Institute for Health Research (NIHR) Health Technology Assessment Programme (grant number NIHR129020). Athena L. Sheppard (Systematic Review Fellowship, grant number NIHR‐RM‐SR‐2017‐08‐012) was funded by the NIHR for this research project. This research was supported by the NIHR Applied Research Collaboration West (NIHR ARC West) at University Hospital Bristol NHS Foundation Trust. The views expressed are those of the author(s) and not necessarily those of the NIHR or the Department of Health and Social Care.

## ETHICAL APPROVAL

Ethical approval was not required.

## Supporting information

Supplementary Material

Supplementary Material

Supplementary Material

## Data Availability

All data and statistical code are available from GitHub (https://github.com/athenasheppard/coeliac‐dta).

## Footnotes

^1^
Although we defined adults as >16 years and children as ≤16 years, cut‐offs for age groups differed between studies in practice, for example studies in children including patients as old as 18 years, studies in adults including patients as young as 15 years.
