# Supplementary material for: Systematic review with meta‐analysis: the accuracy of serological tests to support the diagnosis of coeliac disease
Source: Aliment Pharmacol Ther. 2022 Jan 18;55(5):514–27. doi: 10.1111/apt.16729 (PMC9305515; doi:10.1111/apt.16729)
Supplement: Supplementary file 2 — Supplementary Material [file APT-55-514-s001.pdf]

What is the accuracy of serological testing for diagnosing coeliac disease in adults and children? A systematic review

Athena Sheppard, Penny Whiting, Victoria Corfield, Martha Elwenspoek, Alison Richards, Hayley Jones, Jessica Watson, Peter Gillett, Hazel Everitt

Citation

Athena Sheppard, Penny Whiting, Victoria Corfield, Martha Elwenspoek, Alison Richards, Hayley Jones, Jessica Watson, Peter Gillett, Hazel Everitt. What is the accuracy of serological testing for diagnosing coeliac disease in adults and children? A systematic review. PROSPERO 2019 CRD42019115506 Available from:
https://www.crd.york.ac.uk/prospero/display_record.php?ID=CRD42019115506

Review question

The overall objective is to review the evidence on the diagnostic accuracy of serological tests to identify coeliac disease in adults and children with a clinical suspicion of coeliac disease. We will investigate two research questions to address this objective:

• What is the sensitivity and specificity of serological tests for coeliac disease in adults?

• What is the sensitivity and specificity of serological tests for coeliac disease in children?

Searches

MEDLINE, Embase, Cochrane Library, KSR Evidence (https://ksrevidence.com/) and the Science databases on Web of Science will be searched for relevant studies, combining terms for “antibodies” and “coeliac disease”. Further searches will be carried out to identify grey literature. Ongoing and completed clinical trials will be identified using the WHO International Clinical Trials Registry and the NIH Clinical Trials database, and general internet searches using keywords such as “coeliac” and “serological tests” will be undertaken. The reference lists of recent systematic reviews will also be used as a source of potentially relevant studies. No language or publication restrictions will be applied.

Identified references will be downloaded into Endnote X7 software for further assessment and handling. Rigorous records are maintained as part of the searching process. Individual records within the Endnote reference libraries are tagged with searching information, such as searcher, date searched, database host, database searched, strategy name and iteration, theme or search question. This enables the information specialist to track the origin of each record.

Types of study to be included

Diagnostic accuracy studies. If sufficient diagnostic cohort studies are identified, then case-control studies will be excluded as these have been shown to overestimate test accuracy. Diagnostic cohort studies enrol a group of patients who undergo both serological testing for coeliac disease and the reference standard. Diagnostic case-control studies select groups of patients with and without coeliac disease who then undergo serological testing for coeliac disease.

Condition or domain being studied

Coeliac disease (CD) is an autoimmune disorder in which patients experience gastrointestinal symptoms, including diarrhoea, constipation, vomiting and abdominal pain, triggered by the protein gluten, found in wheat, rye and barley. CD is estimated to affect around 1% of people in the UK, although current evidence suggests that for every case of CD detected in the UK, eight people with CD remain undiagnosed. Currently, the only treatment for CD is lifetime adherence to a gluten free diet. If CD is not diagnosed and treated promptly, damage may be sustained to the surface of the small intestine and difficulty absorbing nutrients may lead to malnutrition, anaemia and/or osteoporosis. In the long-term, untreated CD may lead to a higher risk of serious complications, such as infertility in women, lymphoma and small bowel cancer.

Participants/population
Adults or children at risk of coeliac disease.

Intervention(s), exposure(s)
Any serological test for coeliac disease. Based on the NICE and joint BSPGHAN/Coeliac UK guidelines we expect to include the following tests: immunoglobulin A (IgA) tissue transglutaminase (tTG), immunoglobin G (IgG) tTG, IgA endomysial antibodies (EMA), IgG EMA, IgG deamidated gliadin peptide (DGP) and human leukocyte antigen (HLA)-DQ typing.

Comparator(s)/control
Any reported reference standard; duodenal biopsy is likely to be the most commonly evaluated reference standard.

Main outcome(s)
To estimate the diagnostic accuracy of serological tests to identify coeliac disease in adults and children based on the current literature.

The diagnostic accuracy of a test is measured by the number of true positives, false negatives, false positives and true negatives of the serological (index) test compared to a reference standard indicating the true disease status of each patient. Summary accuracy measures, such as the sensitivity and specificity of each test, are calculated using the count data described above.

Additional outcome(s)
Further test accuracy measures, including the positive and negative predictive value (which additionally depends on the prevalence of the target condition), likelihood ratio and diagnostic odds ratio will additionally be calculated for each test.

Data extraction (selection and coding)

Titles and abstracts identified through electronic database and web searching will be uploaded to Rayyan and independently screened by two reviewers. Reviewers will select studies that they consider potentially relevant for the review. Lists of potentially relevant studies will be compared between reviewers and consensus will be reached through discussion on which records should be obtained as full text. These will be assessed independently by two reviewers to determine whether they meet the inclusion criteria. All papers excluded at this second stage of the screening process will be documented along with the reasons for exclusion. With respect to both screening stages, discrepancies between reviewers will be resolved through discussion or the intervention of a third reviewer.

Data will be extracted using standardised data extraction forms developed in Microsoft Access 2016. Data extraction forms will be piloted on a small sample of papers and adapted as necessary. In order to minimise bias and errors, data extraction will be performed by one reviewer and checked by a second. Disagreements will be resolved through discussion or referral to a third reviewer where necessary.

We will extract the following data from each included paper, where reported:

1. Study characteristics (identifier, study design, study location).

2. Patient characteristics (age, sex, ethnicity, care setting [primary/secondary], inclusion and exclusion criteria).

3. Serological test details (test assessed, threshold for test positivity, name of test, manufacturer, test method/technique).

4. Reference standard details (biopsy procedure and findings).

5. 2×2 data on index test performance (number of true positives, false negatives, false positives, true negatives) at each reported threshold. If a study reports accuracy data on two or more tests, full cross-classified data will be extracted if possible, or separate 2×2 tables of each test otherwise.

6. Accuracy measures (e.g. sensitivity and specificity) will also be extracted and used to check the accuracy of cross-classified tables of test results.

Risk of bias (quality) assessment

Study quality will be assessed using the QUADAS-2 tool which includes domains covering participants, index test, reference standard and flow and timing. The content of the tool will be tailored to the review by adding guidance for interpreting signalling questions as appropriate (in the process of being developed). The tailored QUADAS-2 tool will be trialled in a small number of studies by two reviewers and agreement assessed. If agreement is low, the tool and/or guidelines will be further refined until satisfactory agreement is reached and the tool can be applied to all included studies. If at least one of the domains is rated as “high” the study will be considered at high risk of bias, if all domains are judged as “low” the trial will be considered at low risk of bias, otherwise the trial will be considered at “unclear” risk of bias. The risk of bias assessment will be conducted as part of the data extraction process.

Strategy for data synthesis

A narrative summary of the included studies will be presented. This will include a summary of the characteristics (e.g. study aim, study design, population size, geographical location, year, baseline population characteristics, index test(s) evaluated and reference standard). A detailed commentary on the major methodological problems or biases that affected the studies will also be included.

For each index test, we will perform bivariate random effects meta-analyses of sensitivity and specificity. We will assume binomial likelihoods for the number of true positive and true negative test results. The model accounts for statistical heterogeneity in sensitivity and specificity across studies and between-study correlation in these measures. We will report pooled estimates of sensitivity and specificity with 95% confidence intervals, with 95% confidence and prediction ellipses.

In the absence of study-level covariates, the bivariate random effects model can also produce summary receiver operating characteristic (SROC) curves due to its mathematical equivalence to the hierarchical summary receiver operating characteristic (HSROC) model. If a test is reported at a single threshold for test positivity across studies, summary operating points will be used to measure the test’s accuracy. If a test is reported at differing thresholds across studies, SROC curves showing the trade-off between sensitivity and specificity at the various thresholds will be produced. If 2x2 tables for different thresholds are reported within one study, we will explore novel methods for meta-analysis which extend the bivariate random-effects model to multiple threshold data.

If sufficient data (counts of all possible combinations of test results for patient groups both with and without coeliac disease) are available, novel methods for test comparison data will be explored. These methods jointly meta-analyse evidence on the accuracy of two or more diagnostic tests, allowing for comparisons between index tests (including those which were not directly compared).

Analysis of subgroups or subsets

The analysis will be stratified by age group (children ? 16 years old, adults) and by reason for clinical suspicion/risk status (symptomatic, asymptomatic). Other summary estimates of accuracy, such as likelihood ratios and predictive values, will be estimated from summary estimates of sensitivity and specificity. The calculation of predictive values requires an assumed level of prevalence of the target condition. In the absence of a reliable estimate of the prevalence of CD in patients with a clinical suspicion of the condition (particularly given the variety of signs and symptoms of CD that might arise this suspicion) the positive and negative predictive values will be plotted across a range of possible prevalences.

If sufficient data are available, heterogeneity will be formally investigated by incorporating study-level covariates into the bivariate meta-analysis model. We anticipate that the effects of the following variables will be investigated: study design, ethnicity, presence of gastrointestinal symptoms, existing conditions, family history of autoimmune (including coeliac) disease and IgA deficiency. Other variables considered relevant on further examination of the literature or input from clinical experts may also be considered. The coefficient describing the effect of each variable on sensitivity and specificity will be estimated.

A detailed analysis plan will be produced before the analysis is conducted. Statistical analyses will be performed using Stata (version 15) and RevMan (version 5). Novel methods for test comparison data, if

explored, will be performed in WinBUGS (version 14) and JAGS (version 4).

Contact details for further information

Athena Sheppard
as17849@bristol.ac.uk

Organisational affiliation of the review

NIHR CLAHRC West, University of Bristol
https://clahrc-west.nihr.ac.uk/

Review team members and their organisational affiliations

Miss Athena Sheppard. NIHR CLAHRC West, University of Bristol
Dr Penny Whiting. NIHR CLAHRC West, University of Bristol
Dr Victoria Corfield. NIHR CLAHRC West, University of Bristol
Dr Martha Elwenspoek. NIHR CLAHRC West, University of Bristol
Ms Alison Richards. NIHR CLAHRC West, University of Bristol
Dr Hayley Jones. Bristol Medical School, University of Bristol
Dr Jessica Watson. NIHR CLAHRC West, University of Bristol
Dr Peter Gillett. University of Edinburgh and Royal Hospital for Sick Children
Dr Hazel Everitt. University of Southampton

Type and method of review

Diagnostic, Meta-analysis, Narrative synthesis, Systematic review

Anticipated or actual start date

01 October 2018

Anticipated completion date

30 September 2019

Funding sources/sponsors

This research was funded by the National Institute for Health Research (NIHR) Collaboration for Leadership in Applied Health Research and Care West (NIHR CLAHRC West). The views expressed in this article are those of the author(s) and not necessarily those of the NHS, the NIHR, or the Department of Health and Social Care.

Conflicts of interest

Language

English

Country

England

Stage of review

Review Ongoing

Subject index terms status

Subject indexing assigned by CRD

Subject index terms

Adult; Celiac Disease; Child; Humans; Serologic Tests

Date of registration in PROSPERO

17 January 2019

Date of first submission

16 January 2019

Stage of review at time of this submission

Stage	Started	Completed
Preliminary searches	Yes	No
Piloting of the study selection process	Yes	No
Formal screening of search results against eligibility criteria	No	No
Data extraction	No	No
Risk of bias (quality) assessment	No	No
Data analysis	No	No

The record owner confirms that the information they have supplied for this submission is accurate and complete and they understand that deliberate provision of inaccurate information or omission of data may be construed as scientific misconduct.

The record owner confirms that they will update the status of the review when it is completed and will add publication details in due course.

Versions

17 January 2019

PROSPERO
This information has been provided by the named contact for this review. CRD has accepted this information in good faith and registered the review in PROSPERO. The registrant confirms that the information supplied for this submission is accurate and complete. CRD bears no responsibility or liability for the content of this registration record, any associated files or external websites.
