# Supplementary material for: Systematic review with meta‐analysis: the accuracy of serological tests to support the diagnosis of coeliac disease
Source: Aliment Pharmacol Ther. 2022 Jan 18;55(5):514–27. doi: 10.1111/apt.16729 (PMC9305515; doi:10.1111/apt.16729)
Supplement: Supplementary file 3 — Supplementary Material [file APT-55-514-s002.docx]

# Appendix 1

MEDLINE search strategy. Embase, Cochrane, KSR Evidence and Web of Science were also searched.

1. Celiac Disease/
2. ((coeliac or celiac) adj4 (disease or sprue or syndrome)).tw.
3. ((nontropical or non tropical) adj4 sprue).tw.
4. ((gluten or glutenin or gliadin) adj4 (sensitiv* or hypersensitiv* or intoleran*)).tw.
5. (gluten adj4 enteropath*).tw.
6. or/1-5
7. Serologic Tests/
8. ((serologic or serological) adj4 test*).tw.
9. 7 or 8
10. (endomysi* adj4 antibod*).tw.
11. (immunoglobulin adj4 (endomysi* or anti-endomysi* or antiendomysi* or anti endomysi*)).tw.
12. ((anti-endomysi* or antiendomysi* or anti endomysi*) adj antibod*).tw.
13. ((iga or igg) adj4 (endomysi* or anti-endomysi* or antiendomysi* or anti endomysi*)).tw.
14. (iga-ema or igg-ema).tw.
15. ((EMA or AGA) and antibod*).tw.
16. or/10-15
17. transglutaminases/
18. (((anti-tissue or antitissue or anti tissue) adj4 transglutaminase) and antibod*).tw.
19. ((iga or igg or immunoglobulin) adj4 transglutaminase).tw.
20. ((anti-human or antihuman or anti human or tissue) adj4 transglutaminase adj4 antibod*).tw.
21. (anti-httg or anti-htg or tTg).tw.
22. or/17-21
23. ((gliadin or antigliadin or anti-gliadin or anti gliadin) adj4 antibod*).tw.
24. ((igg or iga or immunoglobulin) adj4 gliadin).tw.
25. ((igg or iga or immunoglobulin) adj4 (antigliadin or anti-gliadin or anti gliadin)).tw.
26. (elisa adj4 test*).tw.
27. Gliadin/ and Immunoglobulins/
28. or/23-27
29. HLA-DQ Antigens/ or HLA-DR3 Antigen/
30. (human adj3 (leukocyte* or leucocyte*) adj3 antigen*).tw.
31. (hla adj3 typing).tw.
32. ((dr3 or hla) adj4 dq2).tw.
33. ((dr4 or hla) adj4 dq8).tw.
34. or/29-33
35. 9 or 16 or 22 or 28 or 34
36. 6 and 35
37. letter/
38. editorial/
39. news/
40. exp historical article/
41. Anecdotes as topic/
42. comment/
43. case report/
44. (letter or comment* or editorial or case report).ti.
45. or/37-44
46. exp animals/ not humans/
47. exp Animals, Laboratory/
48. exp Animal Experimentation/
49. exp Models, Animal/
50. exp rodentia/
51. ((rat or rats or mouse or mice or rodent* or animal* or murine or porcine or feline or canine or dog or dogs or cat or cats or pig or pigs or monkey* or macaque*) not human*).ti.
52. or/46-51
53. 45 or 52
54. 36 not 53

# Appendix 2

QUADAS-2 questions tailored to this review. Signalling questions on applicability were removed as the research question was specific enough that any study meeting the inclusion criteria was applicable by definition.

**Domain 1: Patient selection**

1. Was a consecutive or random sample of patients enrolled?
2. Did the study avoid inappropriate exclusions?
3. Had all patients been on a gluten-containing diet for 6 weeks prior to testing?

**Domain 2: Index test**

1. Were the index test results interpreted without knowledge of the results of the reference standard?
2. If a threshold was used, was it pre-specified?

**Domain 3: Reference standard**

1. Is the reference standard likely to correctly classify the target condition?
2. Were the reference standard results interpreted without knowledge of the results of the index test(s)^[[1]](#footnote-1)^?

**Domain 4: Flow and timing**

1. Was there an appropriate interval between the index test(s) and the reference standard?
2. Were all patients on a gluten-containing diet between index test and reference standard?
3. Did all patients receive a reference standard?
4. Did all patients receive the same reference standard?
5. Were all patients included in the analyses?

# Appendix 3

**Table A1:** Details of included studies.

| **Study, year** | **Patients, n** | **Study population** | | | **Biopsy** | | **Serological test** | | | | | |
| --- | --- | --- | --- | --- | --- | --- | --- | --- | --- | --- | --- | --- |
|  |  | **Prevalence of CD (%)** | **Population** | **Reason for biopsy** | **Threshold for CD diagnosis** | **% biopsied** | **Test** | **TP** | **FN** | **FP** | **TN** | **Threshold for test positivity** |
| Abdollahi et al, 2011.[33] | 134 | 10.4 | Children | NR | NR | 100.0 | IgA tTG  IgA EMA | 4  9 | 10  5 | 6  5 | 114  115 | NR  NR |
| Aberg and Olcen, 2009.[34] | 32 | 75.0 | Children | Symptoms | NR | 100.0 | IgA tTG  IgA/IgG DGP  IgA/IgG tTG/DGP | 21  21  21 | 3  3  3 | 1  1  2 | 7  7  6 | 3.5 or 6 U/mL^1^  20 U/mL  20 U/mL |
| Abrams et al, 2006.[35] | 122 | 83.6 | Adults | Symptoms or risk group | Marsh 3a | 100.0 | IgA tTG | 72 | 30 | 7 | 13 | NR |
| Al Saidi et al, 2013.[36] | 57 | 28.1 | Mixed | Symptoms or risk group | Marsh 1 | 100.0^2^ | IgA EMA | 13 | 3 | 0 | 41 | 10 U/mL |
| Ali et al, 2012.[37] | 174 | 12.6 | Children | NR | NR | 100.0 | IgA tTG | 13 | 9 | 4 | 148 | 100 U/mL |
| Alonso et al, 2009.[38] | 149 | 68.5 | Children | NR | NR | 100.0 | IgA tTG | 91 | 11 | 2 | 45 | 12 U/mL |
| Ashorn et al, 2008.[39] | 242 | 55.4 | Mixed | Symptoms | NR | 100.0 | IgA tTG | 127 | 7 | 10 | 98 | NR |
| Atkinson et al, 1997.[40] | 66 | 33.3 | Mixed | NR | NR | 100.0^2^ | IgA EMA | 21 | 1 | 16 | 28 | 1:10 |
| Barada et al, 2014.[41] | 999 | 1.8 | Adults | Symptoms or risk group | Marsh 3a | 100.0^2^ | IgA tTG  IgA/IgG tTG/DGP | 13  13 | 5  5 | 16  25 | 965  955 | NR  NR |
| Barker et al, 2005.[42] | 103 | 56.3 | Children | Symptoms or risk group | Marsh 2 | 100.0 | IgA tTG | 55 | 3 | 10 | 35 | 20 U/mL |
| Basso et al, 2001.[43] | 72 | 52.8 | Children | NR | NR | 100.0 | IgA tTG  IgA EMA | 28  37 | 3  1 | 0  1 | 20  33 | 5.5 U/mL  NR |
| Bayram et al, 2015.[44] | 66 | 68.2 | Mixed | NR | Marsh 1 | 100.0 | IgA tTG | 42 | 3 | 19 | 2 | NR |
| Bhatnagar et al, 2005.[45] | 259 | 24.3 | Children | Symptoms | Marsh 3a | 100.0 | IgA EMA | 56 | 7 | 88 | 108 | 1:10 |
| Bishop et al, 2018.[46] | 103 | 83.5 | Children | Symptoms or risk group | Marsh 2 | 100.0 | IgA tTG  IgA EMA  IgA/IgG DGP | 84  86  85 | 2  0  1 | 13  12  5 | 4  5  12 | 21 U/mL  NR  20 U/mL |
| Bowron et al, 2000.[47] | 112  454 | 6.3  5.9 | Adults  Children | NR  NR | NR  NR | NR  NR | IgA EMA  IgA EMA | 6  24 | 1  3 | 4  12 | 101  415 | NR  NR |
| Bozzato et al, 2011.[48] | 500 | 46.4 | Children | NR | NR | 100.0 | IgA/IgG tTG  IgA/IgG tTG/DGP | 219  220 | 13  12 | 38  29 | 230  239 | 45.1 U/mL  33.7 U/mL |
| Burginwolff et al, 1990.[49] | 548 | 61.5 | Children | Symptoms | NR | 100.0 | IgA EMA | 304 | 33 | 4 | 207 | NR |
| Camarero et al, 1997.[50] | 71 | 54.9 | Children | Symptoms or risk group | NR | 100.0 | IgA EMA | 37 | 2 | 6 | 26 | 1:40 |
| Carroccio et al, 2002.[51] | 207 | 11.6 | Adults | Symptoms | NR | 100.0 | IgA tTG  IgA EMA | 24  24 | 0  0 | 6  0 | 177  183 | 7 U/mL  NR |
| Carroccio et al, 2006.[52] | 273 | 70.0 | Mixed | Symptoms | Marsh 2 | 100.0 | IgA tTG  IgA EMA | 162  159 | 29  32 | 4  1 | 78  81 | 7 U/mL  NR |
| Chan et al, 2001.[53] | 77 | 13.0 | Children | Symptoms or risk group | NR | 100.0 | IgA tTG  IgA EMA | 8  8 | 2  2 | 4  2 | 63  65 | NR  1:10 |
| Clouzeau et al, 2007.[54] | 106 | 50.0 | Children | Symptoms | Marsh 3a | 100.0 | IgA tTG | 53 | 0 | 3 | 50 | NR |
| Corrao et al, 1994.[55] | 93 | 47.3 | Adults | NR | NR | 100.0 | IgA EMA | 43 | 1 | 0 | 49 | 1:5 |
| Dahlbom et al, 2016.[56] | 242 | 20.7 | Children | NR | NR | 62.0 | IgA tTG  IgG tTG  IgA/IgG tTG  IgA/IgG DGP  IgA/IgG tTG/DGP | 44  42  48  47  46 | 6  8  2  3  4 | 1  3  1  7  1 | 191  189  191  185  191 | 3 U/mL  3 U/mL  6 U/mL  20 U/mL  3 U/mL |
| Dahle et al, 2010.[57] | 172 | 43.6 | Adults | Symptoms | Marsh 3a | 100.0 | IgA tTG  IgA EMA  IgA/IgG DGP  IgA/IgG tTG/DGP | 57  46  65  68 | 18  29  10  7 | 5  0  4  19 | 92  97  93  78 | 5 U/mL  1:5  20 U/mL  20 U/mL |
| Del Rosario et al, 1998.[58] | 46 | 10.9 | Children | Symptoms or risk group | NR | 100.0^2^ | IgA EMA | 5 | 0 | 0 | 41 | NR |
| Diamanti et al, 2006.[59] | 277 | 58.1 | Children | Symptoms or risk group | Marsh 1 | 100.0^2^ | IgA tTG | 161 | 0 | 25 | 91 | 4 U/mL |
| Dickey et al, 1997.[60] | 318 | 9.7 | Mixed | Symptoms or risk group | NR | 100.0 | IgA EMA | 27 | 4 | 0 | 287 | 1:5 |
| Donaldson et al, 2007.[61] | 117 | 47.9 | Children | Symptoms or risk group | Marsh 3a | 100.0 | IgA tTG  IgA EMA | 48  48 | 8  8 | 8  12 | 53  49 | 20 U/mL  1:10 |
| Dutta et al, 2010.[62] | 92 | 19.6 | Mixed | Symptoms | Marsh 1 | 100.0 | IgA tTG | 14 | 4 | 8 | 66 | 15 U/mL |
| El Zanati et al, 2019.[63] | 168 | 11.3 | NR | NR | NR | 100.0 | IgA tTG | 16 | 3 | 10 | 139 | NR |
| Elliott et al, 2009.[64] | 31 | 54.8 | NR | NR | NR | 100.0^2^ | IgA tTG  IgA/IgG DGP | 15  12 | 2  5 | 6  1 | 8  13 | NR  NR |
| Emami et al, 2008.[65] | 350 | 6.0 | Mixed | Symptoms or risk group | Marsh 3a | 100.0 | IgA tTG | 8 | 13 | 6 | 323 | 10 U/mL |
| Eremic et al, 2012.[66] | 50 | 8.0 | Adults | NR | NR | 100.0 | IgA tTG | 3 | 1 | 0 | 46 | NR |
| Feighery et al, 1998.[67] | 441 | 22.0 | Mixed | Symptoms | NR | 100.0 | IgA EMA | 84 | 13 | 4 | 340 | NR |
| Fernandez et al, 2005.[68] | 122 | 33.6 | Mixed | Symptoms or risk group | Marsh 1 | 100.0 | IgA tTG  IgA EMA | 41  40 | 0  1 | 2  2 | 79  79 | 3 U/mL  1:5 |
| Ford, 2009.[69] | 58 | 36.2 | NR | NR | NR | 100.0 | IgA tTG  IgA/IgG DGP | 20  18 | 1  3 | 12  1 | 25  36 | NR  NR |
| Gandolfi et al, 2001.[70] | 33 | 15.2 | Children | Symptoms | Marsh 3a | 100.0^2^ | IgA EMA | 2 | 3 | 0 | 28 | 1:5 |
| Gidrewicz et al, 2015.[71] | 1011 | 33.7 | Children | Symptoms or risk group | Marsh 3a | 100.0^2^ | IgA tTG | 338 | 3 | 99 | 571 | 20 U/mL |
| Gonzalez et al, 2012.[72] | 171 | 28.2 | NR | NR | NR | 100.0 | IgA DGP  IgG DGP | 37  35 | 11  11 | 8  10 | 114  115 | 19.9 U/mL  19.9 U/mL |
| Grodzinsky et al, 1995.[73] | 100 | 27.0 | Children | NR | NR | 100.0 | IgA EMA | 21 | 6 | 1 | 72 | NR |
| Hadithi et al, 2007.[74] | 463 | 3.5 | Adults | Symptoms or risk group | Marsh 3a | 100.0 | IgA tTG  IgA EMA | 13  13 | 3  3 | 4  4 | 443  443 | NR  NR |
| Hashmi et al, 2016.[75] | 60 | 63.3 | Children | Symptoms | Marsh 3a | 100.0 | IgA tTG | 33 | 5 | 4 | 18 | 7 U/mL |
| Hojsak et al, 2012.[76] | 59 | 79.7 | Children | NR | NR | 100.0 | IgA tTG  IgA/IgG EMA  IgA/IgG DGP | 46  44  31 | 1  2  0 | 6  1  7 | 6  10  2 | 20 U/mL  1:2.5  20 U/mL |
| Hollen et al, 2016.[77] | 138 | 64.5 | Children | Symptoms or risk group | NR | 100.0 | IgA tTG  IgA EMA | 81  62 | 8  5 | 4  6 | 45  47 | 7 U/mL  1:10 |
| Holmes et al, 2017.[78] | 270 | 92.6 | Adults | NR | Marsh 1 | 100.0 | IgA tTG | 242 | 8 | 20 | 0 | 5 U/mL |
| Hopper et al, 2007.[79] | 109 | 16.5 | NR | Symptoms | NR | 100.0 | IgA tTG | 16 | 2 | 3 | 88 | NR |
| Hopper et al, 2008.[80] | 2000 | 3.9 | Adults | Symptoms | Marsh 3a | 100.0 | IgA tTG  IgA EMA | 70  67 | 7  10 | 175  37 | 1748  1886 | 15 U/mL  NR |
| Iiritano et al, 2013.[81] | 46 | 34.8 | NR | NR | Marsh 3a | 100.0 | IgA tTG | 15 | 1 | 1 | 29 | NR |
| Iwanczak et al, 2003.[82] | 93 | 38.7 | Children | Symptoms or risk group | NR | 100.0^2^ | IgA tTG  IgG tTG  IgA EMA | 30  21  35 | 6  15  1 | 4  3  7 | 53  54  50 | NR  NR  NR |
| Jarmi et al, 2010.[83] | 169 | 38.5 | Adults | Symptoms or risk group | NR | 100.0 | IgA tTG  IgA EMA  IgA/IgG DGP | 55  56  56 | 10  9  9 | 1  1  2 | 103  103  102 | 12 U/mL  NR  20 U/mL |
| Javaeed et al, 2015.[84] | 121 | 11.6 | Adults | Symptoms | Marsh 3a | 100.0 | IgA tTG | 11 | 3 | 2 | 105 | NR |
| Johnston et al, 2003.[85] | 92 | 31.5 | Adults | NR | NR | 100.0 | IgA tTG  IgA EMA | 25  26 | 4  3 | 10  1 | 53  62 | 25 U/mL  1:5 |
| Jora et al, 2017.[86] | 52 | 26.9 | Children | Symptoms | Marsh 3a | 100.0^2^ | IgA tTG | 14 | 0 | 35 | 3 | 20 U/mL |
| Kabatova et al, 2017.[87] | 181 | 90.6 | Children | Symptoms | Marsh 3a | 100.0 | IgA tTG  IgA EMA | 110  120 | 54  44 | 4  7 | 13  10 | NR  NR |
| Kashif et al, 2017.[88] | 178 | 53.9 | Mixed | NR | Marsh 2 | 100.0 | IgA tTG  IgG tTG | 46  39 | 50  57 | 0  18 | 82  64 | 10 U/mL  10 U/mL |
| Kocna et al, 2002.[89] | 112 | 44.6 | NR | Symptoms or risk group | NR | 100.0^2^ | IgA tTG  IgA EMA | 44  34 | 6  16 | 22  4 | 40  58 | 10 U/mL  NR |
| Kotze et al, 2003.[90] | 47 | 61.7 | Mixed | Symptoms | NR | 100.0 | IgA EMA | 29 | 0 | 11 | 7 | 1:2.5 |
| Kurien et al, 2013.[91] | 523 | 32.3 | Adults | NR | Marsh 3a | 100.0 | IgA tTG | 152 | 17 | 60 | 294 | 15 U/mL |
| Kutty et al, 2014.[92] | 544 | 54.2 | Children | NR | NR | 100.0 | IgA tTG | 288 | 7 | 117 | 132 | NR |
| Lau et al, 2018.[93] | 1000 | 4.1 | Adults | Symptoms | Marsh 3a | 100.0 | IgA tTG  IgA EMA | 32  29 | 9  12 | 36  2 | 923  957 | NR  NR |
| Leon et al, 2001.[94] | 238 | 36.1 | NR | Symptoms or risk group | NR | 84.9 | IgA tTG  IgA EMA | 85  85 | 1  1 | 1  2 | 151  150 | NR  1:10 |
| Lindquist et al, 1994.[95] | 77 | 54.5 | Children | Symptoms | NR | 100.0 | IgA EMA | 41 | 1 | 3 | 32 | 1:2.5 |
| Llorente et al, 2004.[96] | 125 | 48.8 | Mixed | Symptoms or risk group | NR | 100.0 | IgA tTG  IgA EMA | 61  43 | 0  1 | 3  2 | 61  40 | 3.5 U/mL  1:5 |
| Lock et al, 2004.[97] | 109 | 28.4 | NR | NR | NR | 100.0 | IgA tTG  IgA EMA | 31  28 | 0  3 | 26  1 | 52  25 | NR  NR |
| Longarini et al, 2014.[98] | 161 | 39.1 | Adults | NR | Marsh 3a | 100.0 | IgA tTG  IgG DGP | 60  60 | 3  3 | 2  0 | 96  98 | 20 U/mL  20 U/mL |
| McClements et al, 2013.[99] | 44 | 34.1 | NR | NR | NR | 100.0^2^ | IgA tTG | 15 | 0 | 1 | 28 | NR |
| McMillan et al, 1991.[100] | 96 | 29.2 | Adults | Symptoms | NR | 100.0 | IgA EMA  IgG EMA | 25  11 | 3  17 | 0  1 | 68  67 | 1:20  1:20 |
| Mendez et al, 1998.[101] | 17 | 58.8 | Children | Symptoms | Marsh 3a | 100.0 | IgA EMA | 8 | 2 | 4 | 3 | 1:20 |
| Mooney et al, 2014.[102] | 523 | 22.4 | Adults | Symptoms or risk group | Marsh 3a | 100.0 | IgA tTG  IgA EMA | 106  98 | 11  19 | 67  10 | 339  396 | 15 U/mL  NR |
| Mooney et al, 2015.[103] | 508 | 13.4 | Adults | Symptoms or risk group | Marsh 3a | 100.0 | IgA tTG  IgA EMA | 62  61 | 6  7 | 55  15 | 385  425 | 15 U/mL  NR |
| Moreno et al, 2007.[104] | 391 | 2.6 | NR | Symptoms | Marsh 2 | 100.0 | IgA tTG  IgA DGP  IgG DGP  IgA/IgG DGP  IgA AAA | 8  9  8  8  5 | 2  1  2  2  5 | 14  13  3  5  33 | 367  368  378  376  348 | NR  NR  NR  NR  NR |
| Mubarak et al, 2011.[105] | 212 | 51.4 | Children | Symptoms or risk group | Marsh 3a | 100.0 | IgA tTG  IgA EMA  IgA DGP  IgG DGP  IgA/IgG tTG/DGP | 105  107  93  100  107 | 4  2  16  9  2 | 16  32  12  17  40 | 87  71  91  86  63 | 10 U/mL  NR  20 U/mL  20 U/mL  20 U/mL |
| Mubarak et al, 2012.[106] | 183 | 65.6 | Children | Symptoms or risk group | Marsh 2 | 100.0 | IgA tTG  IgA EMA | 116  116 | 4  4 | 14  20 | 49  43 | 10 U/mL  NR |
| Murphy et al, 2001.[107] | 67 | 86.6 | NR | NR | Marsh 3a | 100.0^2^ | IgA tTG  IgA EMA | 48  58 | 10  0 | 7  4 | 1  5 | NR  NR |
| Nguyen et al, 2019.[108] | 272 | 86.0 | Children | NR | Marsh 2 | 100.0 | IgA tTG | 154 | 80 | 1 | 37 | 40 U/mL |
| Niveloni et al, 2006.[109] | 122 | 46.7 | NR | NR | Marsh 2 | 100.0 | IgA tTG | 54 | 3 | 2 | 63 | NR |
| Niveloni et al, 2007.[110] | 141 | 42.6 | Adults | Symptoms or risk group | Marsh 2 | 100.0 | IgA tTG  IgA DGP  IgG DGP  IgA/IgG DGP  IgA AAA | 57  59  58  59  52 | 3  1  2  1  8 | 2  5  0  1  4 | 79  76  81  80  77 | 20 U/mL  20 U/mL  20 U/mL  20 U/mL  25 U/mL |
| Novogrudsky et al, 2004.[111] | 145 | 82.8 | NR | Symptoms or risk group | NR | 100.0 | IgA tTG | 83 | 37 | 10 | 15 | NR |
| Olen et al,[112] 2012 | 530 | 52.1 | Children | Symptoms or risk group | Marsh 2 | 100.0 | IgA tTG  IgA/IgG DGP | 259  172 | 17  16 | 36  164 | 218  56 | 3 or 7 U/mL^1^  16 or 20 U/mL^1^ |
| Pallav et al,[113] 2014 | 172 | 70.9 | Adults | Symptoms or risk group | Marsh 1 | 100.0^2^ | IgA tTG | 43 | 79 | 16 | 34 | 19 U/mL |
| Panetta et al,[114] 2011 | 155 | 69.7 | Children | Symptoms | Marsh 3a | 100.0 | IgA tTG  IgA EMA | 104  103 | 4  5 | 4  2 | 43  45 | 8 U/mL  1:5 |
| Parizade et al,[115] 2009 | 116 | 73.3 | Children | Symptoms or risk group | Marsh 1 | 100.0 | IgA tTG  IgG tTG  IgA/IgG EMA  IgA/IgG DGP | 80  55  81  81 | 5  30  4  4 | 4  2  8  11 | 27  29  23  20 | 5 U/mL  7 U/mL  1:5  20 U/mL |
| Pearce et al, 2002.[116] | 104 | 24.0 | NR | Symptoms | NR | 100.0 | IgA EMA | 21 | 4 | 0 | 79 | NR |
| Pettei and Levine, 2009.[117] | 46 | 63.0 | Children | NR | NR | 100.0 | IgG tTG | 9 | 20 | 0 | 17 | NR |
| Poddar et al, 2008.[118] | 306 | 58.8 | Children | Symptoms | NR | 100.0 | IgA tTG | 178 | 2 | 9 | 117 | 4 U/mL |
| Polanco et al, 2001.[119] | 260 | 45.4 | Children | NR | NR | 100.0 | IgA tTG | 109 | 9 | 4 | 138 | 12 U/mL |
| Pretto et al, 2019.[120] | 33 | 75.8 | Children | NR | Marsh 3a | 100.0 | IgA tTG  IgA EMA  IgG DGP | 23  21  23 | 2  4  2 | 1  0  1 | 7  8  7 | 10 U/mL  1:5  10 U/mL |
| Previtali et al, 2018.[121] | 549  223 | 36.2  64.6 | Adults  Children | NR  NR | Marsh 2  Marsh 2 | 100.0  100.0 | IgA tTG  IgA tTG | 193  144 | 6  0 | 6  1 | 344  78 | NR  NR |
| Rabbani et al, 2015.[122] | 117 | 60.7 | Children | Symptoms or risk group | Marsh 3a | 100.0 | IgA tTG | 66 | 5 | 22 | 24 | 17 U/mL |
| Rahmati et al, 2014.[123] | 159 | 83.6 | Mixed | Symptoms | Marsh 3a | 100.0 | IgA tTG | 131 | 2 | 16 | 10 | 89.5 U/mL |
| Reeves et al, 2006.[124] | 254 | 10.2 | Mixed | Symptoms or risk group | NR | 100.0 | IgA tTG  IgG tTG  IgA/IgG tTG | 23  22  24 | 3  4  2 | 37  25  39 | 191  203  189 | 2.8 U/mL  18.9 U/mL  7.8 U/mL |
| Roca et al, 2019.[125] | 445 | 52.1 | Children | Symptoms or risk group | Marsh 2 | 56.9 | IgA tTG  IgA EMA | 229  228 | 3  4 | 15  1 | 198  212 | 7 U/mL  1:5 |
| Russo et al, 1999.[126] | 95 | 25.3 | Children | NR | NR | 100.0 | IgA EMA | 11 | 13 | 3 | 68 | NR |
| Saneian and Gorgani, 2012.[127] | 57 | 42.1 | Children | Symptoms | Marsh 2 | 100.0 | IgA tTG  IgA EMA | 8  12 | 10  12 | 0  8 | 15  25 | NR  NR |
| Santaolalla et al, 2008.[128] | 312 | 13.5 | Mixed | Symptoms or risk group | Marsh 3a | 100.0 | IgA tTG | 39 | 3 | 14 | 256 | 2 U/mL |
| Sayed et al, 2012.[129] | 65 | 21.5 | Adults | Symptoms | Marsh 2 | 100.0 | IgA tTG  IgG tTG  IgA EMA  IgA DGP  IgG DGP | 9  10  11  12  13 | 5  4  3  2  1 | 6  2  0  4  0 | 45  49  51  47  51 | 10 U/mL  10 U/mL  1:5  10 U/mL  10 U/mL |
| Scoglio et al, 2003.[130] | 50  131 | 68.0  76.3 | Adults  Children | Symptoms or risk group  Symptoms or risk group | Marsh 3a  Marsh 3a | 100.0  100.0 | IgA tTG  IgA EMA  IgA tTG  IgA EMA | 34  33  99  95 | 0  1  1  5 | 3  2  9  4 | 13  14  22  27 | NR  NR  NR  NR |
| Srinivas et al, 2014.[131] | 752 | 11.7 | NR | Symptoms or risk group | Marsh 1 | 100.0 | IgA tTG  IgA EMA | 73  70 | 15  18 | 29  5 | 635  659 | 10 U/mL  NR |
| Sugai et al, 2010.[132] | 679 | 11.8 | Adults | Symptoms | Marsh 3a | 100.0 | IgA tTG  IgA DGP  IgG DGP  IgA/IgG DGP  IgA/IgG tTG/DGP  IgA AAA | 73  76  72  74  77  64 | 7  4  8  6  3  16 | 15  26  5  22  66  47 | 584  573  594  577  533  552 | 20 U/mL  20 U/mL  20 U/mL  20 U/mL  20 U/mL  25 U/mL |
| Swallow et al, 2013.[133] | 756 | 3.0 | Adults | NR | Marsh 3a | 100.0 | IgA tTG  IgA EMA | 21  19 | 2  4 | 77  7 | 656  726 | 15 U/mL  NR |
| Tesei et al, 2003.[134] | 426 | 58.7 | Adults | Symptoms or risk group | Marsh 2 | 100.0 | IgA tTG  IgA EMA | 225  214 | 25  36 | 9  0 | 167  176 | 7 U/mL  1:5 |
| Tronconi et al, 2010.[135] | 102 | 76.5 | Children | NR | NR | 100.0 | IgA tTG  IgG tTG | 75  76 | 3  2 | 10  7 | 14  17 | NR  NR |
| Valdimarsson et al, 1996.[136] | 144 | 13.2 | Adults | Symptoms | Marsh 3a | 100.0 | IgA EMA | 14 | 5 | 0 | 125 | 1:10 |
| Vargas Perez et al, 2005.[137] | 218 | 69.7 | Children | Symptoms or risk group | NR | 100.0^2^ | IgA EMA | 134 | 18 | 3 | 63 | NR |
| Vogelsang et al, 1995.[138] | 102 | 48.0 | Adults | Symptoms | NR | 100.0 | IgA EMA | 49 | 0 | 0 | 53 | NR |
| Volta et al, 2010.[139] | 144 | 33.3 | Adults | Symptoms or risk group | Marsh 3a | 100.0 | IgA tTG  IgA EMA | 45  44 | 3  4 | 10  7 | 86  89 | 16 U/mL  1:5 |
| Weile et al, 2002.[140] | 233 | 17.2 | Children | Symptoms | NR | 100.0 | IgA tTG | 16 | 24 | 1 | 192 | 10 U/mL |
| Whyte et al, 2001.[141] | 48 | 16.7 | Adults | NR | NR | 100.0 | IgA tTG | 8 | 0 | 9 | 31 | 20 U/mL |
| Wildfang et al, 1992.[142] | 70 | 34.3 | Children | Symptoms or risk group | NR | 100.0 | IgA EMA | 24 | 0 | 0 | 46 | 1:10 |
| Wolf et al, 2017.[30] | 890 | 60.1 | Children | Symptoms or risk group | NR | 100.0^2^ | IgA tTG  IgA EMA  IgG DGP | 514  503  413 | 21  25  122 | 36  20  21 | 319  325  334 | 20 U/mL  1:10  25 U/mL |
| Yiannakou et al, 1998.[143] | 144 | 23.6 | NR | Symptoms or risk group | NR | 100.0 | IgA EMA | 32 | 2 | 2 | 108 | NR |
| Zanini et al, 2012.[144] | 945 | 63.1 | Adults | Symptoms or risk group | Marsh 3a | 100.0 | IgA tTG | 543 | 53 | 64 | 285 | 7, 8 or 16 U/mL^1^ |

CD, coeliac disease; TP, true positives; FN, false negatives; FP, false positives; TN, true negatives; NR, not reported

^------------------------------------------------------------------------------------------------------------^

^1^ More than one threshold used for test positivity due to differences in manufacturer

^2^ Additional patients were recruited to study, but did not undergo biopsy and are excluded from analysis

# Appendix 4

**Table A2:** Risk of bias assessments using QUADAS-2.

| **Study, year** | **2x2 data**  **(where more than one set per study)** | **Risk of bias** | | | |
| --- | --- | --- | --- | --- | --- |
|  |  | **Patient selection** | **Reference standard** | **Index test** | **Flow and timing** |
| Abdollahi et al, 2011.[33] | IgA tTG  IgA EMA | ?  ? | ?  ? | ?  ? | ?  ? |
| Aberg and Olcen, 2009.[34] | IgA tTG  IgA/IgG DGP  IgA/IgG tTG/DGP | ?  ?  ? | ☹  ☹  ☹ | ☺  ☺  ☺ | ☹  ☹  ☹ |
| Abrams et al, 2006.[35] |  | ☺ | ☺ | ? | ? |
| Al Saidi et al, 2013.[36] |  | ? | ☹ | ☺ | ☹ |
| Ali et al, 2012.[37] |  | ☺ | ☹ | ? | ? |
| Alonso et al, 2009.[38] |  | ? | ☹ | ? | ? |
| Ashorn et al, 2008.[39] |  | ? | ☹ | ? | ? |
| Atkinson et al, 1997.[40] |  | ? | ☹ | ☺ | ☺ |
| Barada et al, 2014.[41] | IgA tTG  IgA/IgG tTG/DGP | ?  ? | ☺  ☺ | ?  ? | ?  ? |
| Barker et al, 2005.[42] |  | ☺ | ☺ | ☺ | ☺ |
| Basso et al, 2001.[43] | IgA tTG  IgA EMA | ?  ? | ☹  ☹ | ☺  ? | ☹  ☺ |
| Bayram et al, 2015.[44] |  | ☹ | ☹ | ? | ? |
| Bhatnagar et al, 2005.[45] |  | ☺ | ☹ | ☺ | ? |
| Bishop et al, 2018.[46] | IgA tTG  IgA EMA  IgA/IgG-DGP | ☺  ☺  ☺ | ☺  ☺  ☺ | ☺  ?  ☺ | ☺  ☺  ☺ |
| Bowron et al, 2000.[47] | Adults  Children | ?  ? | ☹  ☹ | ?  ? | ☹  ☹ |
| Bozzato et al, 2011.[48] | IgA/IgG tTG  IgA/IgG tTG/DGP | ?  ? | ☹  ☹ | ☹  ☹ | ☹  ☹ |
| Burginwolff et al, 1990.[49] |  | ? | ☹ | ? | ? |
| Camarero et al, 1997.[50] |  | ☹ | ☺ | ☺ | ☹ |
| Carroccio et al, 2002.[51] | IgA tTG  IgA EMA | ☺  ☺ | ☺  ☺ | ☺  ? | ☺  ☺ |
| Carroccio et al, 2006.[52] | IgA tTG  IgA EMA | ☺  ☺ | ☺  ☺ | ☺  ? | ?  ? |
| Chan et al, 2001.[53] | IgA tTG  IgA tTG | ?  ? | ☺  ☺ | ☺  ☺ | ?  ? |
| Clouzeau et al, 2007.[54] |  | ? | ☹ | ? | ? |
| Corrao et al, 1994.[55] |  | ? | ☹ | ☺ | ? |
| Dahlbom et al, 2016.[56] | IgA tTG  IgG tTG  IgA/IgG tTG  IgA/IgG DGP  IgA/IgG tTG/DGP | ☺  ☺  ☺  ☺  ☺ | ☹  ☹  ☹  ☹  ☹ | ☺  ☺  ☹  ☹  ☹ | ☹  ☹  ☹  ☹  ☹ |
| Dahle et al, 2010.[57] | IgA tTG  IgA EMA  IgA/IgG DGP  IgA/IgG tTG/DGP | ☺  ☺  ☺  ☺ | ☺  ☺  ☺  ☺ | ☺  ☺  ☺  ☺ | ☺  ☺  ☺  ☺ |
| Del Rosario et al, 1998.[58] |  | ☺ | ☹ | ? | ☹ |
| Diamanti et al, 2006.[59] |  | ? | ☹ | ☺ | ☹ |
| Dickey et al, 1997.[60] |  | ? | ☹ | ☺ | ? |
| Donaldson et al, 2007.[61] | IgA tTG  IgA EMA | ?  ? | ☺  ☺ | ☺  ☺ | ?  ? |
| Dutta et al, 2010.[62] |  | ? | ☹ | ☺ | ? |
| El Zanati et al, 2019.[63] |  | ? | ☹ | ? | ☹ |
| Elliott et al, 2009.[64] | IgA tTG  IgA/IgG-DGP | ?  ? | ☹  ☹ | ?  ? | ☹  ☹ |
| Emami et al, 2008.[65] |  | ☺ | ☺ | ☺ | ? |
| Eremic et al, 2012.[66] |  | ? | ☹ | ? | ? |
| Feighery et al, 1998.[67] |  | ? | ☹ | ? | ? |
| Fernandez et al, 2005.[68] | IgA tTG  IgA EMA | ?  ? | ☹  ☹ | ☺  ☺ | ?  ? |
| Ford, 2009.[69] | IgA tTG  IgA/IgG DGP | ☺  ☺ | ☹  ☹ | ?  ? | ?  ? |
| Gandolfi et al, 2001.[70] |  | ☺ | ☺ | ☺ | ☹ |
| Gidrewicz et al, 2015.[71] |  | ☺ | ☹ | ☺ | ☺ |
| Gonzalez et al, 2012.[72] | IgA DGP  IgG DGP | ?  ? | ☹  ☹ | ☺  ☺ | ?  ? |
| Grodzinsky et al, 1995.[73] |  | ☺ | ☺ | ? | ? |
| Hadithi et al, 2007.[74] | IgA tTG  IgA EMA | ☺  ☺ | ☺  ☺ | ?  ? | ?  ? |
| Hashmi et al, 2016.[75] |  | ☺ | ☹ | ☺ | ☺ |
| Hojsak et al, 2012.[76] | IgA tTG  IgA/IgG EMA  IgA/IgG DGP | ?  ?  ? | ☹  ☹  ☹ | ☺  ☺  ☺ | ?  ?  ☹ |
| Hollen et al, 2016.[77] | IgA tTG  IgA EMA | ?  ? | ☹  ☹ | ?  ? | ☺  ☺ |
| Holmes et al, 2017.[78] |  | ? | ☹ | ☺ | ? |
| Hopper et al, 2007.[79] |  | ? | ☹ | ? | ? |
| Hopper et al, 2008.[80] | IgA tTG  IgA EMA | ☺  ☺ | ☹  ☹ | ☺  ? | ☺  ☺ |
| Iiritano et al, 2013.[81] |  | ? | ☹ | ? | ? |
| Iwanczak et al, 2003.[82] | IgA tTG  IgG tTG  IgA EMA | ☺  ☺  ☺ | ☹  ☹  ☹ | ?  ?  ? | ☹  ☹  ☹ |
| Jarmi et al, 2010.[83] | IgA tTG  IgA EMA  IgA/IgG DGP | ☺  ☺  ☺ | ☹  ☹  ☹ | ☺  ?  ☺ | ?  ?  ? |
| Javaeed et al, 2015.[84] |  | ? | ☺ | ☺ | ? |
| Johnston et al, 2003.[85] | IgA tTG  IgA EMA | ?  ? | ☹  ☹ | ☺  ☺ | ?  ? |
| Jora et al, 2017.[86] |  | ☺ | ☺ | ☺ | ☹ |
| Kabatova et al, 2017.[87] | IgA tTG  IgA EMA | ?  ? | ☹  ☹ | ?  ☹ | ?  ? |
| Kashif et al, 2017.[88] | IgA tTG  IgG tTG | ?  ? | ☹  ☹ | ☺  ☺ | ?  ? |
| Kocna et al, 2002.[89] | IgA tTG  IgA EMA | ? ? | ☹  ☹ | ☺  ? | ☹  ☹ |
| Kotze et al, 2003.[90] |  | ☹ | ☹ | ☺ | ☹ |
| Kurien et al, 2013.[91] |  | ? | ☹ | ☺ | ☺ |
| Kutty et al, 2014.[92] |  | ? | ☹ | ? | ☹ |
| Lau et al, 2018.[93] | IgA tTG  IgA EMA | ☺  ☺ | ☺  ☺ | ?  ? | ?  ? |
| Leon et al, 2001.[94] | IgA tTG  IgA EMA | ?  ? | ☹  ☹ | ☺  ☺ | ☹  ☹ |
| Lindquist et al, 1994.[95] |  | ☹ | ☹ | ☺ | ☹ |
| Llorente et al, 2004.[96] | IgA tTG  IgA EMA | ?  ? | ☺  ☺ | ☺  ☹ | ☹  ? |
| Lock et al, 2004.[97] | IgA tTG  IgA EMA | ☹  ☹ | ☹  ☹ | ?  ? | ?  ☹ |
| Longarini et al, 2014.[98] | IgA tTG  IgG DGP | ?  ? | ☹  ☹ | ☺  ☺ | ?  ? |
| McClements et al, 2013.[99] |  | ? | ☹ | ? | ☹ |
| McMillan et al, 1991.[100] | IgA EMA  IgG EMA | ☺  ☺ | ☺  ☺ | ☺  ☺ | ☺  ☺ |
| Mendez et al, 1998.[101] |  | ? | ☹ | ☺ | ? |
| Mooney et al, 2014.[102] | IgA tTG  IgA EMA | ?  ? | ☺  ☺ | ☺  ? | ?  ? |
| Mooney et al, 2015.[103] | IgA tTG  IgA EMA | ☺  ☺ | ☺  ☺ | ☺  ? | ☺  ☺ |
| Moreno et al, 2007.[104] | IgA tTG  IgA DGP  IgG DGP  IgA/IgG DGP  IgA AAA | ?  ?  ?  ? ? | ☹  ☹  ☹  ☹  ☹ | ?  ?  ?  ?  ? | ?  ?  ?  ?  ? |
| Mubarak et al, 2011.[105] | IgA tTG  IgA EMA  IgA DGP  IgG DGP  IgA/IgG tTG/DGP | ☺  ☺  ☺  ☺  ☺ | ☺  ☺  ☺  ☺  ☺ | ☺  ?  ☺  ☺  ☺ | ?  ?  ?  ?  ? |
| Mubarak et al, 2012.[106] | IgA tTG  IgA EMA | ☺  ☺ | ☺  ☺ | ☺  ? | ?  ? |
| Murphy et al, 2001.[107] | IgA tTG  IgA EMA | ?  ? | ☺ ☺ | ?  ? | ?  ? |
| Nguyen et al, 2019.[108] |  | ? | ☹ | ☺ | ? |
| Niveloni et al, 2006.[109] |  | ? | ☹ | ? | ☹ |
| Niveloni et al, 2007.[110] | IgA tTG  IgA DGP  IgG DGP  IgA/IgG DGP  IgA AAA | ☺  ☺  ☺  ☺  ☺ | ☺  ☺  ☺  ☺  ☺ | ☺  ☺  ☺  ☺  ☺ | ☺  ☺  ☺  ☺  ☺ |
| Novogrudsky et al, 2004.[111] |  | ? | ☹ | ? | ? |
| Olen et al, 2012.[112] | IgA tTG  IgA/IgG DGP | ?  ? | ☹  ☹ | ☺  ☺ | ?  ? |
| Pallav et al, 2014.[113] |  | ☺ | ☹ | ☺ | ☹ |
| Panetta et al, 2011.[114] | IgA tTG  IgA EMA | ☹  ☹ | ☺  ☺ | ☺  ☺ | ?  ? |
| Parizade et al, 2009.[115] | IgA tTG  IgG tTG  IgA/IgG EMA  IgA/IgG DGP | ?  ?  ?  ? | ☺  ☺  ☺  ☺ | ☺  ☺  ☺  ☺ | ?  ?  ?  ? |
| Pearce et al, 2002.[116] |  | ☹ | ☹ | ? | ? |
| Pettei and Levine, 2009.[117] |  | ? | ☹ | ? | ? |
| Poddar et al, 2008.[118] |  | ☹ | ☹ | ☺ | ☹ |
| Polanco et al, 2001.[119] |  | ☹ | ☺ | ☹ | ☹ |
| Pretto et al, 2019.[120] | IgA tTG  IgA EMA  IgG DGP | ?  ?  ? | ☹  ☹  ☹ | ☺  ☺  ☺ | ?  ?  ? |
| Previtali et al, 2018.[121] | Adults  Children | ☺  ☺ | ☺  ☺ | ☺  ☺ | ☺  ☺ |
| Rabbani et al, 2015.[122] |  | ☺ | ☹ | ☺ | ☺ |
| Rahmati et al, 2014.[123] |  | ? | ☹ | ☹ | ? |
| Reeves et al, 2006.[124] | IgA tTG  IgG tTG  IgA/IgG tTG | ?  ?  ? | ☺  ☺  ☺ | ☹  ☹  ☹ | ?  ?  ? |
| Roca et al, 2019.[125] | IgA tTG  IgA EMA | ☹  ☹ | ☹  ☹ | ☺  ☺ | ☹  ☹ |
| Russo et al, 1999.[126] |  | ☺ | ☹ | ☺ | ☺ |
| Saneian and Gorgani, 2012.[127] | IgA tTG  IgA EMA | ☹  ☹ | ☹  ☹ | ?  ? | ☹  ? |
| Santaolalla et al, 2008.[128] |  | ☺ | ☺ | ☺ | ☺ |
| Sayed et al, 2012.[129] | IgA tTG  IgG tTG  IgA EMA  IgA DGP  IgG DGP | ☺  ☺  ☺  ☺  ☺ | ☹  ☹  ☹  ☹  ☹ | ☺  ☺  ☺  ☺  ☺ | ☺  ☺  ☺  ☺  ☺ |
| Scoglio et al, 2003.[130] | Adults, IgA tTG  Adults, IgA EMA  Children, IgA tTG  Children, IgA EMA | ?  ?  ?  ? | ☺  ☺  ☺  ☺ | ?  ?  ?  ? | ?  ?  ?  ? |
| Srinivas et al, 2014.[131] | IgA tTG  IgA EMA | ☺  ☺ | ☹  ☹ | ☺  ? | ☹  ☹ |
| Sugai et al, 2010.[132] | IgA tTG  IgA DGP  IgG DGP  IgA/IgG DGP  IgA/IgG tTG/DGP  IgA AAA | ☹  ☹  ☹  ☹  ☹  ☹ | ☺  ☺  ☺  ☺  ☺  ☺ | ☺  ☺  ☺  ☺  ☺  ☺ | ?  ?  ?  ?  ?  ? |
| Swallow et al, 2013.[133] | IgA tTG  IgA EMA | ☺  ☺ | ☹  ☹ | ☺  ? | ☺  ☺ |
| Tesei et al, 2003.[134] | IgA tTG  IgA EMA | ?  ? | ☺  ☺ | ☺  ☺ | ?  ? |
| Tronconi et al, 2010.[135] | IgA tTG  IgG tTG | ?  ? | ☹  ☹ | ?  ? | ?  ? |
| Valdimarsson et al, 1996.[136] |  | ? | ☺ | ☺ | ☹ |
| Vargas Perez et al, 2005.[137] |  | ☹ | ☹ | ? | ☹ |
| Vogelsang et al, 1995.[138] |  | ☺ | ☹ | ☺ | ☺ |
| Volta et al, 2010.[139] | IgA tTG  IgA EMA | ?  ? | ☹  ☹ | ☺  ☺ | ?  ? |
| Weile et al, 2002.[140] |  | ☹ | ☺ | ? | ☹ |
| Whyte et al, 2001.[141] |  | ? | ☹ | ☺ | ? |
| Wildfang et al, 1992.[142] |  | ? | ☺ | ☺ | ☺ |
| Wolf et al, 2017.[30] | IgA tTG  IgA EMA  IgG DGP | ☺  ☺  ☺ | ☺  ☺  ☺ | ☺  ☺  ☺ | ☺  ☺  ☺ |
| Yiannakou et al, 1998.[143] |  | ? | ☹ | ? | ? |
| Zanini et al, 2012.[144] |  | ☺ | ☹ | ☺ | ? |

☺ = low risk of bias

☹ = high risk of bias

? = unclear risk of bias

# Appendix 5


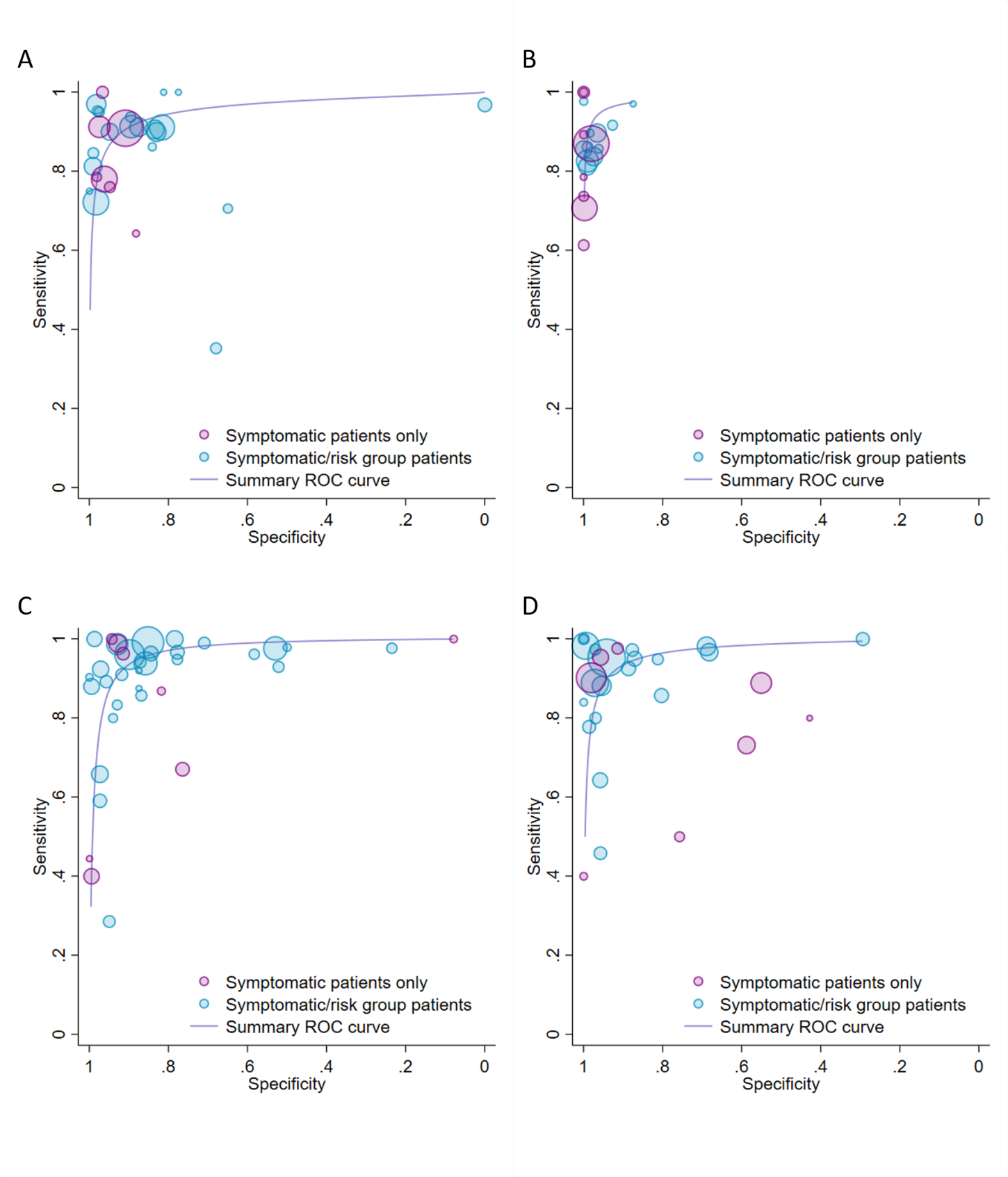


**Figure A1:** Study estimates of test sensitivity and specificity in adults and children plotted in receiver operating characteristic (ROC) space, stratified by reason for biopsy. Summary ROC curves are estimated from a meta-analysis of all data, across thresholds. [A] Adults, IgA tTG; [B] Adults, IgA EMA; [C] Children, IgA tTG; [D] Children, IgA EMA.


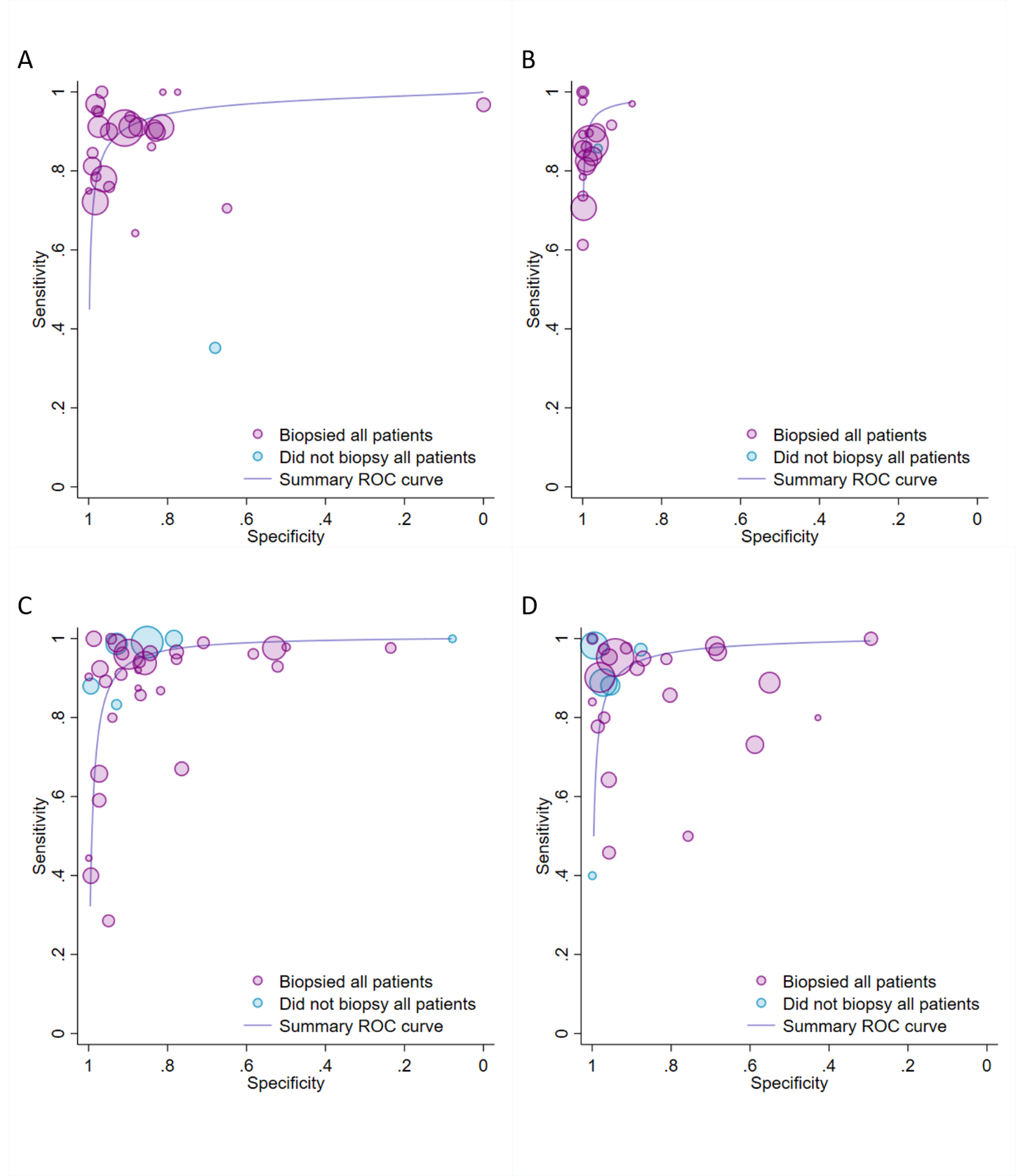


**Figure A2:** Study estimates of test sensitivity and specificity in adults and children plotted in receiver operating characteristic (ROC) space, stratified by whether all patients underwent biopsy. Summary ROC curves are estimated from a meta-analysis of all data, across thresholds. [A] Adults, IgA tTG; [B] Adults, IgA EMA; [C] Children, IgA tTG; [D] Children, IgA EMA.


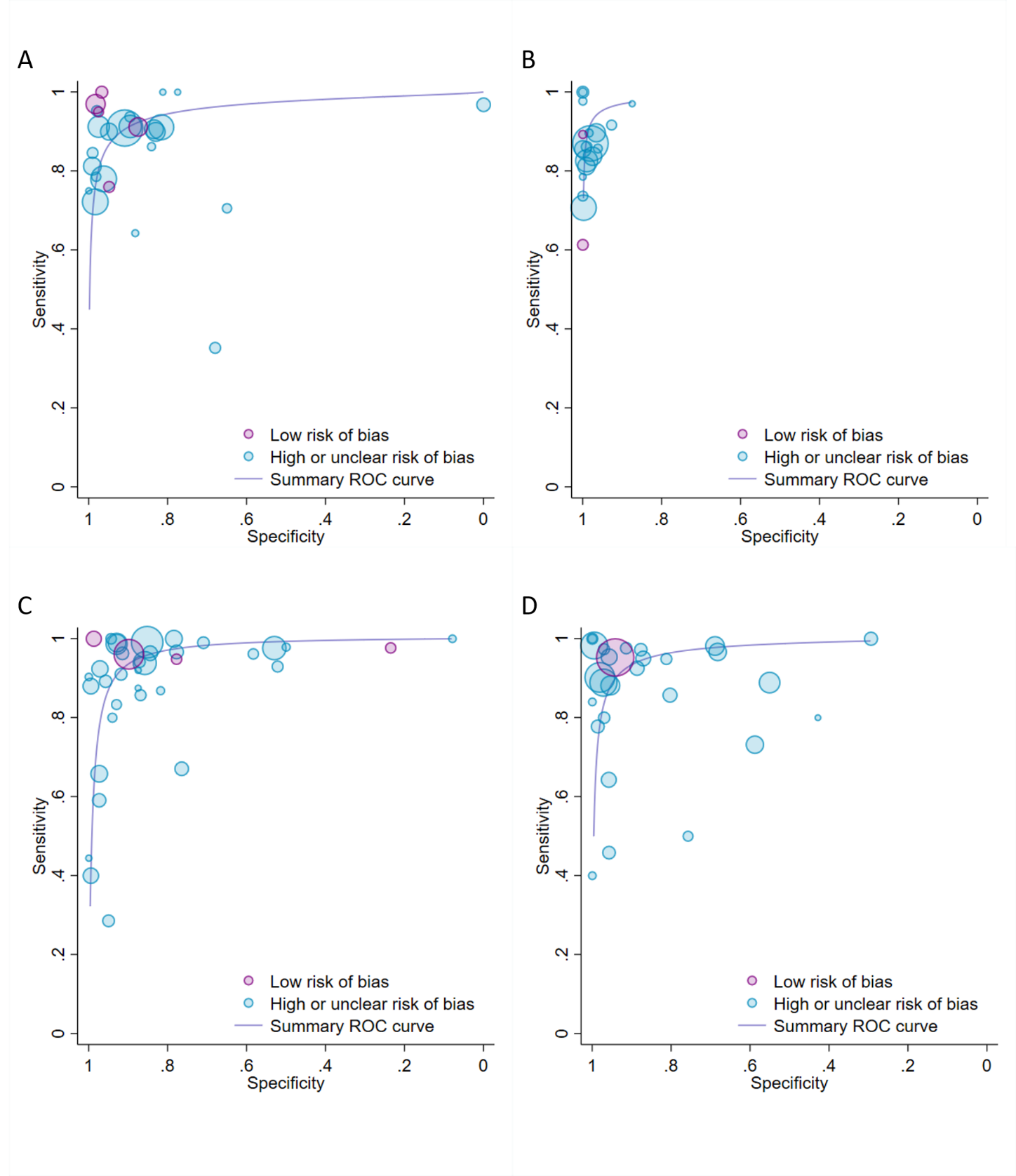


**Figure A3:** Study estimates of test sensitivity and specificity in adults and children plotted in receiver operating characteristic (ROC) space, stratified by risk of bias. Summary ROC curves are estimated from a meta-analysis of all data, across thresholds. [A] Adults, IgA tTG; [B] Children, IgA tTG.

1. Where it was not explicitly stated that reference standard (biopsy) results were interpreted without knowledge of index test (serology) results, risk of bias was assumed high rather than unclear. Without a conscious effort to blind pathologists to serology or other clinical characteristics of the patient, it is likely that they would have had access to these results. [↑](#footnote-ref-1)
